# Macrophage migration inhibitory factor (MIF) acetylation protects neurons from ischemic injury

**DOI:** 10.1038/s41419-022-04918-2

**Published:** 2022-05-18

**Authors:** Jin-Xia Hu, Wei-Jing Ma, Li-Ying He, Cong-Hui Zhang, Cheng Zhang, Yan Wang, Chao-Nan Chen, Da-Yong Shen, Hui-Min Gao, Rui-Ru Guo, Qian-Qian Ning, Xin-Chun Ye, Gui-Yun Cui, Lei Li

**Affiliations:** 1 Institute of Stroke Research and Department of Neurology, the Affiliated Hospital of Xuzhou Medical University, Xuzhou Medical University, Xuzhou, Jiangsu China; 2 ShanghaiTech University, Shanghai, China; 3 Department of Neurology, Affiliated municipal Hospital of Xuzhou Medical University; Xuzhou Medical University, Xuzhou, Jiangsu China; 4 University of Chinese Academy of Sciences, Beijing, China; 5 Cancer Institute, Xuzhou Medical University, Xuzhou, Jiangsu China

**Keywords:** Cell death in the nervous system, Stroke

## Abstract

Ischemia-induced neuronal death leads to serious lifelong neurological deficits in ischemic stroke patients. Histone deacetylase 6 (HDAC6) is a promising target for neuroprotection in many neurological disorders, including ischemic stroke. However, the mechanism by which HDAC6 inhibition protects neurons after ischemic stroke remains unclear. Here, we discovered that genetic ablation or pharmacological inhibition of HDAC6 reduced brain injury after ischemic stroke by increasing macrophage migration inhibitory factor (MIF) acetylation. Mass spectrum analysis and biochemical results revealed that HDAC6 inhibitor or aspirin treatment promoted MIF acetylation on the K78 residue. MIF K78 acetylation suppressed the interaction between MIF and AIF, which impaired MIF translocation to the nucleus in ischemic cortical neurons. Moreover, neuronal DNA fragmentation and neuronal death were impaired in the cortex after ischemia in MIF K78Q mutant mice. Our results indicate that the neuroprotective effect of HDAC6 inhibition and aspirin treatment results from MIF K78 acetylation; thus, MIF K78 acetylation may be a therapeutic target for ischemic stroke and other neurological diseases.

## Introduction

Acetylation of lysine (K) residues is an important posttranslational modification that functions in regulating many cellular processes. Acetylation was initially discovered on histones, and histone acetylation is associated with chromatin remodeling and transcriptional regulation [1–3]. In addition to histone acetylation, thousands of nonhistone acetylated proteins have been identified in the regulation of cellular functions [4–7]. A member of the histone deacetylase family, histone deacetylase 6 (HDAC6), localizes to the cytosol and regulates many cellular processes by deacetylating nonhistone proteins, including α-tubulin, cortactin, Hsp90, and peroxiredoxin [8–11]. A growing body of evidence indicates that HDAC6 inhibition is a promising target for neurological diseases [12–18]; however, the mechanism by which HDAC6 is involved in these neurological disorders remains unknown.

Acetylation of α-tubulin K40, a well-known substrate of HDAC6, is implicated in neuroprotection after HDAC6 inhibition in many neurological diseases [13, 14, 16]. Whether α-tubulin K40 acetylation alone is responsible for neurological diseases remains to be verified. Tubulin acetyltransferase (ATAT1)-deficient mice, which have no α-tubulin K40 acetylation, show deficits in neuronal migration and axon branching [19, 20] without developing any neurological deficits [21]. Therefore, the identification of novel HDAC6 substrates may contribute to understanding how HDAC6 participates in the development of neurological diseases.

Ischemic brain injury is the leading cause of death and adult disability worldwide. Neuronal death due to ischemia results in parts of the brain becoming dysfunctional, leading to face drooping, arm weakness, and slurred speech in ischemic stroke patients. Ischemia may elevate the production of reactive oxygen species (ROS) and induce DNA damage in neurons [22]. Neuronal DNA damage during ischemia leads to excessive activation of poly (ADP-ribose) (PAR) polymerase-1 (PARP-1), producing PAR polymers. Inhibition or genetic deletion of PARP-1 protects neurons from death after ischemic stroke [23]. Furthermore, PAR polymers induce binding of macrophage migration inhibitory factor (MIF) to apoptosis-inducing factor (AIF) in ischemic neurons, resulting in translocation of MIF to the nucleus [24, 25]; nuclear MIF causes large-scale DNA fragmentation and neuronal death [25, 26].

In this study, we found that MIF acetylation was increased by treatment with an HDAC6 inhibitor or aspirin. We further demonstrated that MIF K78 acetylation attenuated the interaction between MIF and AIF, resulting in impairment of MIF nuclear translocation and therefore protecting neurons from ischemic stroke. Our results indicate that increased MIF K78 acetylation contributes to the neuroprotective effect of HDAC6 inhibitors in ischemic stroke.

## Results

### HDAC6 suppression protects against ischemia-induced brain injury

HDAC6 is expressed in the mouse brain [27]. We dissected different brain regions and analyzed HDAC6 expression by immunoblotting in mice. As shown in Fig. S1A, B, HDAC6 was highly expressed in the cortex and cerebellum. Moreover, HDAC6 was predominantly expressed in cultured neurons other than astrocytes or microglia (Fig. S1C), and immunostaining revealed that HDAC6 was costained with the neuronal marker NeuN in the cortex (Fig. S1D). To determine whether HDAC6 expression is implicated in stroke, we induced ischemic stroke in the cortex, where HDAC6 is highly expressed, using the photothrombotic model. HDAC6 protein was augmented in the penumbra regions 3, 5, and 7 days following ischemia (Fig. S1E, F). Quantitative PCR (qPCR) also showed a time-dependent increase in *Hdac6* mRNA after ischemia (Fig. S1G), suggesting increased *Hdac6* transcription in the penumbra region of the cortex after ischemic stroke.

Previous studies have shown that postischemic treatment with tubastatin A, an HDAC6 inhibitor, reduced brain injury in the middle cerebral artery occlusion (MCAO) model in rats [18]. Using HDAC6 mutant mice, we studied whether HDAC6 deficiency regulates neuronal death after ischemia. HDAC6 mutant mice, confirmed by PCR genotyping and immunoblotting (Fig. S2A, B), appeared normal (Fig. S2C, D). There was little difference between HDAC6 mutant mice and wild-type (WT) littermates in brain size and weight (Fig. S2E, F). Furthermore, we observed no significant difference in neuron count or main brain structure between HDAC6 mutants and WT mice (Fig. S2G). Next, we induced photothrombotic ischemia in HDAC6 mutants and WT mice. Severe brain infarction was observed on Day 3 after ischemia in WT mice (Fig. S2H). Triphenyl tetrazolium chloride (TTC) staining revealed that the infarct volume in the cortex was significantly reduced in HDAC6 mutant mice (7.09 ± 0.59%) compared with WT mice (12.67 ± 0.93%) (Fig. S2H, I). We further performed various stroke damage-associated behavioral assays, including the modified neurological severity score (mNSS), adhesive-removal test, and foot fault test. HDAC6 mutant mice showed significantly better performance than WT mice in these behavioral tests (Fig. S2J–L), suggesting that HDAC6 mutant mice have reduced neurological deficits and better motor-sensory function after ischemia.

### MIF is a novel substrate of HDAC6

We next investigated which protein acetylation was regulated by HDAC6 and involved in neuronal death after ischemia. To explore novel substrates of HDAC6, we used mass spectrometry to identify any hyperacetylated proteins in the HDAC6-deficient cortex. A total of 218 proteins were found to be acetylated in this tissue (Table S1). We then performed Metascape analysis and gene set enrichment analysis (GSEA) to determine which pathways the acetylated proteins were enriched in and identified two pathways that play important roles in neuronal death in ischemic stroke, regulation of intrinsic apoptotic signaling pathway (GO:2001242) and regulation of cell death pathway (Fig. S3A, B). Among the proteins in the cell death pathway, we selected the MIF protein for further study because MIF serves as a nuclease to induce DNA fragmentation and neuronal death in ischemia [25].

**Fig. 1:**
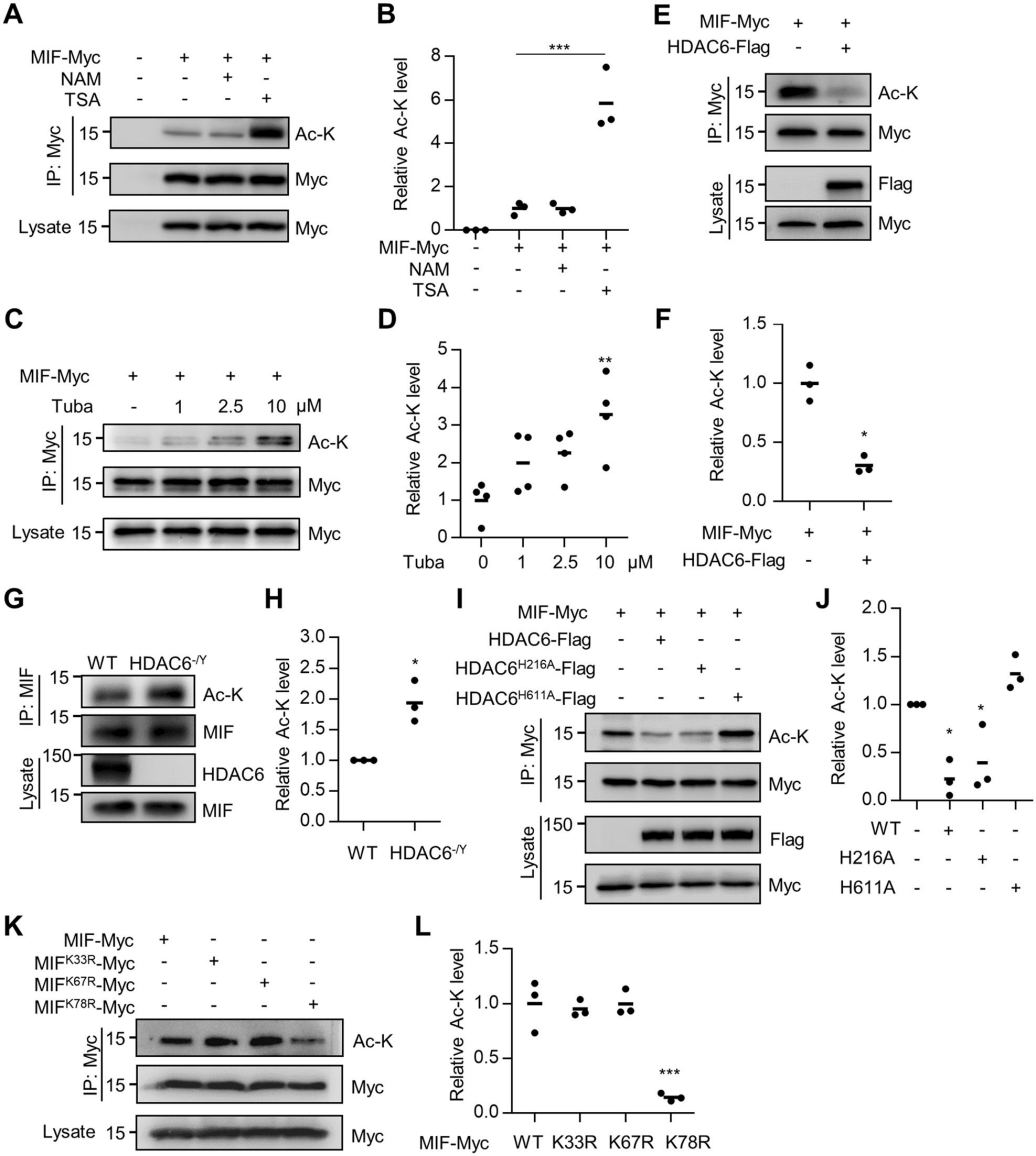
Deacetylation of MIF protein by HDAC6. **A** Increased MIF acetylation in cells treated with HDAC inhibitors. HEK293T cells were transfected with MIF-Myc and treated with nicotinamide (NAM) or trichostatin A (TSA). MIF protein was immunoprecipitated with anti-Myc antibody and probed with anti-Ac-K antibody to reveal acetylated MIF protein. **B** Quantification of MIF acetylation showed in **A**. Data shown were independent points and mean; one-way ANOVA; *n* = 3; ***p < 0.001. **C** Increased MIF acetylation in tubastatin A-treated cells. HEK293T cells were transfected with MIF-Myc and treated with HDAC6 inhibitor tubastatin A at indicated concentrations. MIF protein was immunoprecipitated with anti-Myc antibody and probed with anti-Ac-K antibody. **D** Quantification of MIF acetylation showed in **C**. Data shown were independent points and mean; one-way ANOVA; *n* = 4; **p < 0.01. **E** Decreased MIF acetylation in HDAC6-overexpressing cells. HEK293T cells were transfected with MIF-Myc with or without HDAC6-Flag. MIF protein was immunoprecipitated with anti-Myc antibody and probed with anti-Ac-K antibody. **F** Quantification of MIF acetylation showed in **E**. Data shown were independent points and mean; paired *t*-test; *n* = 3; **p < 0.01. **G** Increased MIF acetylation in HDAC6 mutant mice. The lysates from WT and HDAC6 mutant cortex were immunoprecipitated with anti-MIF antibody and probed with anti-Ac-K antibody. **H** Quantification of MIF acetylation showed in **G**. Data shown were independent points and mean; paired *t*-test; *n* = 3; *p < 0.05. **I** The second deacetylase domain of HDAC6 is required for MIF deacetylation. HDAC6-Flag, two deacetylase domain mutants (H216A and H611A), and MIF-Myc were transfected into HEK293T cells. MIF was immunoprecipitated with anti-Myc antibody and probed with anti-Ac-K antibody. **J** Quantification of MIF acetylation showed in **I**. Data shown were independent points and mean; one-way ANOVA; *n* = 3; *p < 0.05. **K** K78 of MIF is the major acetylation site. MIF-Myc, K33R-Myc, K67R-Myc, and K78R-Myc were transfected into HEK293T cells. MIF was immunoprecipitated with anti-Myc antibody and probed with anti-Ac-K antibody. **L** Quantification of MIF acetylation is shown in **K**. Data shown were independent points and mean; one-way ANOVA; *n* = 3; *F* = 27.39, *p* < 0.001; ***p < 0.001.

To determine whether MIF can be acetylated, HEK293T cells were transfected with Myc-tagged MIF and treated with an inhibitor of sirtuin family deacetylases (nicotinamide; NAM) or the pan-HDAC inhibitor trichostatin A (TSA) [28]. MIF protein was purified by anti-Myc antibody, and acetylated MIF was revealed by the anti-Ac-K antibody. As shown in Fig. 1A, B, MIF acetylation was dramatically augmented in TSA-treated cells, suggesting that MIF acetylation is regulated by HDAC family deacetylases. Furthermore, the acetylation level of MIF was markedly increased in HEK293T cells treated with the HDAC6-specific inhibitor tubastatin A (Fig. 1C, D), indicating that HDAC6 may deacetylate MIF. To further test this hypothesis, we conducted the following experiments. First, MIF acetylation was decreased in HEK293T cells cotransfected with HDAC6 (Fig. 1E, F). Second, MIF acetylation was increased in the cortex of HDAC-deficient mice compared with WT littermates (Fig. 1G, H). Third, we generated two HDAC6 mutants, H216A and H611A, which disrupt the enzymatic activity in the first and second deacetylase domains, respectively [29, 30]. MIF acetylation was impaired when cotransfected with WT HDAC6 or the first deacetylase domain mutant HDAC6^H216A^; in contrast, MIF acetylation was not altered by the second deacetylase domain mutant HDAC6^H611A^ (Fig. 1I, J), suggesting that the second deacetylase domain of HDAC6 is required for regulating MIF acetylation. Finally, we determined which K residue was acetylated. The MIF protein contains three K residues; we, therefore, replaced each K with arginine (R) to generate three K-to-R mutants. The K-to-R mutation on K78 largely reduced MIF acetylation, whereas mutations on other K residues had little effect (Fig. 1K, L), suggesting that K78 is the major acetylation site of MIF. Together, these observations indicate that HDAC6 may regulate MIF acetylation on the K78 residue.

### Enhanced acetylation of MIF by aspirin

**Fig. 2:**
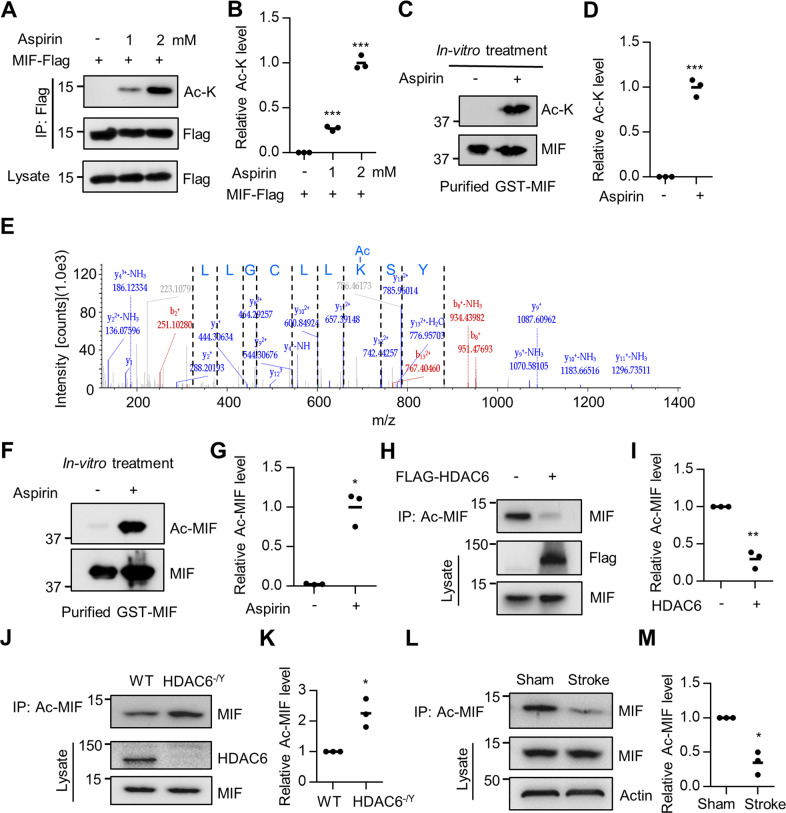
Enhanced MIF acetylation with aspirin treatment. **A** Increased MIF acetylation in cells treated with aspirin. HEK293T cells were transfected with MIF-Flag and treated with aspirin at indicated concentrations. MIF protein was immunoprecipitated with anti-Myc antibody and probed with anti-Ac-K antibody. **B** Quantification of MIF acetylation showed in (**A**). Data shown were independent points and mean; one-way ANOVA; *n* = 3; ***p < 0.001. **C** Incubation of recombinant GST-MIF protein with aspirin (1 mM), and immunoblot with anti-Ac-K antibody. **D** Quantification of MIF acetylation showed in (**C**). Data shown were independent points and mean; paired *t*-test; *n* = 3; ***p < 0.001. **E** Representative K78-acetylated peptides of MIF were analyzed by mass spectrometry. HEK293T cells were transfected with MIF-Flag and treated with aspirin (2 mM). The MIF protein was immunoprecipitated with an anti-Flag antibody and acetylated peptides were analyzed by mass spectrometry. The y ion peaks are shown in blue, and the b ion peaks are shown in red. **F** Incubation of recombinant GST-MIF protein with aspirin at indicated concentrations, and immunoblot with MIF K78 acetylation antibody. **G** Quantification of MIF K78 acetylation is shown in (**F**). Data shown were independent points and mean; paired *t*-test; *n* = 3; *p < 0.05. **H** Decreased MIF K78 acetylation in HDAC6-overexpressing cells. HEK293T cells were transfected with Flag-HDAC6. Acetylated MIF protein was immunoprecipitated with anti-Ac-MIF antibody and probed with anti-MIF antibody. **I** Quantification of MIF K78 acetylation shown in **H**. Data shown were independent points and mean; paired *t*-test; *n* = 3; **p < 0.01. **J** Increased MIF K78 acetylation in HDAC6 mutant cortex. Lysates from WT and HDAC6 mutant cortex were immunoprecipitated with anti-Ac-MIF antibody and probed with anti-MIF antibody. **K** Quantification of MIF K78 acetylation is shown in (**J**). Data shown were independent points and mean; paired *t*-test; *n* = 3; *p < 0.05. **L** Reduced MIF K78 acetylation in the penumbra regions of the cortex. Lysates of the cortex from the control and ischemia-induced mice were immunoprecipitated with anti-Ac-MIF antibody and probed with anti-MIF antibody. **M** Quantification of MIF K78 acetylation is shown in (**L**). Data shown were independent points and mean; paired *t*-test; *n* = 3; *p < 0.05.

Aspirin has been shown to reduce the risk of recurrent stroke[31]. The effect of aspirin is mainly attributed to its antiplatelet actions by inhibition of cyclooxygenase (COX)-dependent pathways [32]. However, it has been shown to exert neuroprotective effects through various mechanisms [33–35]. In addition, aspirin has been shown to acetylate multiple proteins to modulate their cellular functions [36, 37]. Thus, we tested whether aspirin could acetylate MIF and found that MIF acetylation was increased in a dose-dependent manner in aspirin-treated HEK293T cells (Fig. 2A, B). Incubation of purified GST-tagged MIF (GST-MIF) with aspirin led to remarkable MIF acetylation detected by anti-Ac-K antibody (Fig. 2C, D), suggesting that aspirin may directly transfer the acetyl group to GST-MIF in vitro. Additionally, we examined MIF acetylation in aspirin-treated HEK293T cells by performing liquid chromatography–mass spectrometry (LC–MS) with immunoprecipitated MIF protein. Consistent with our prior results, we found that aspirin-induced acetylation of MIF protein was primarily identified on K78 (Fig. 2E). We further generated a site-specific anti-K78 acetylation antibody using the peptide RNYSK (78)^AC^LLC to detect acetylated MIF K78. Aspirin-induced GST-MIF acetylation was revealed by the anti-K78 acetylation antibody (Fig. 2F, G). Together, these results indicate that aspirin can induce MIF acetylation on the K78 residue.

**Fig. 3:**
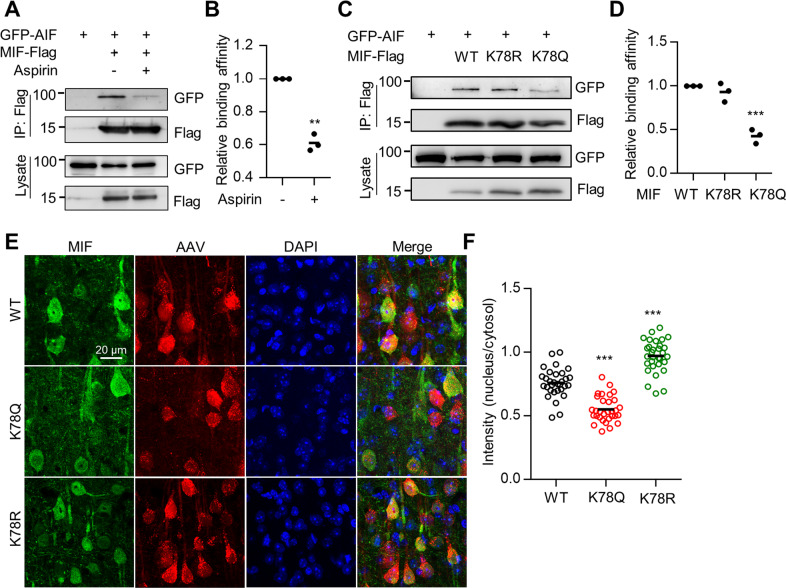
MIF K78 acetylation impaired MIF interaction with AIF and nuclear translocation. **A** Reduced interaction between MIF and AIF after aspirin treatment. HEK293T cells were transfected with GFP-AIF and MIF-Flag. MIF protein was immunoprecipitated with anti-Flag antibody and the resulting complex was probed with anti-GFP antibody. **B** Quantification of AIF interaction with MIF shown in (**A**). Data shown were independent points and mean; *n* = 3; paired *t*-test; **p < 0.01. **C** Acetylation of MIF K78 impaired binding to AIF. HEK293T cells were transfected with MIF-Flag, K78R-Flag, K78Q-Flag, and GFP-AIF. MIF protein was immunoprecipitated with an anti-Flag antibody, and the resulting complex was probed with an anti-GFP antibody. **D** Quantification of AIF interaction with MIF shown in (**C**). Data shown were independent points and mean; one-way ANOVA; *n* = 3; ***p < 0.001. **E** Acetylation of MIF K78 impaired MIF translocation to the nucleus after ischemia. AAV-MIF-2A-mCherry, AAV-K78Q-2A-mCherry, and AAV-K78R-2A-mCherry were injected into the cortex and ischemia was induced. Brain sections were stained with anti-MIF antibody (green). **F** Quantification analysis of MIF intensity in the nucleus/cytosol. Data shown were mean ± SEM; one-way ANOVA; *n* = 16 cells were randomly selected from three mice; ***p < 0.001.

Using the anti-K78 acetylation antibody, we found that MIF K78 acetylation was reduced in HEK293T cells transfected with HDAC6 (Fig. 2H, I) and that MIF K78 acetylation was increased in the HDAC6-deficient cortex (Fig. 2J, K), suggesting that HDAC6 deacetylates MIF on the K78 residue. Furthermore, MIF K78 acetylation was decreased in the penumbra regions of the cortex 3 days after ischemic stroke (Fig. 3L, M), likely resulting from increased HDAC6 expression in the cortex after ischemia.

### MIF acetylation impairs AIF interaction and nuclear translocation

MIF is involved in DNA fragmentation during ischemia-induced neuronal death [25]. To determine whether MIF acetylation is involved in cell death, we treated Neuro2a cells with methylnitronitrosoguanidine (MNNG) to induce PARP-1-dependent cell death [25]. Propidium iodide (PI) staining revealed that both the HDAC6 inhibitor tubastatin A and aspirin attenuated MNNG-induced cell death (Fig. S4A, B). In addition, we found that tubastatin A and aspirin inhibited MNNG-induced DNA fragmentation in Neuro2a cells (Fig. S4C–F). These results indicate that MIF acetylation induced by HDAC6 inhibition or aspirin attenuates MNNG-induced cell death in vitro.

During ischemic stroke, MIF interacts with apoptosis-inducing factor (AIF) to translocate from the cytosol to the nucleus to cause DNA fragmentation and neuronal death [25]. We next examined whether MIF acetylation regulates the interaction between MIF and AIF. We observed MIF-Flag binding with GFP-AIF; this interaction between AIF and MIF was reduced in aspirin-treated cells (Fig. 3A, B). Furthermore, we used the K-to-Q and K-to-R mutants to mimic acetylation and acetylation-deficient MIF, respectively [5, 38]. The MIF K78Q mutant showed decreased binding to AIF, whereas the K78R mutant was still associated with AIF (Fig. 3C, D). Together, these results indicate that MIF acetylation on K78 impairs binding to AIF. To examine whether MIF translocation to the nucleus is also impaired by acetylation after ischemia, adenosine-associated viruses (AAV) containing WT, K78Q, and K78R MIF were administered into the cortex. After 2 weeks, ischemia was induced in the virus-injected cortical regions. The confocal images revealed that WT MIF protein localized in both the cytosol and nucleus in cortical neurons after ischemia. In comparison, MIF K78Q showed attenuated nuclear MIF intensity, while MIF K78R showed increased nuclear MIF intensity (Fig. 3E, F). These results indicate that MIF acetylation on K78 attenuates translocation to the nucleus through impaired interaction with AIF.

### MIF acetylation on K78 reduces ischemia-induced brain injury

**Fig. 4:**
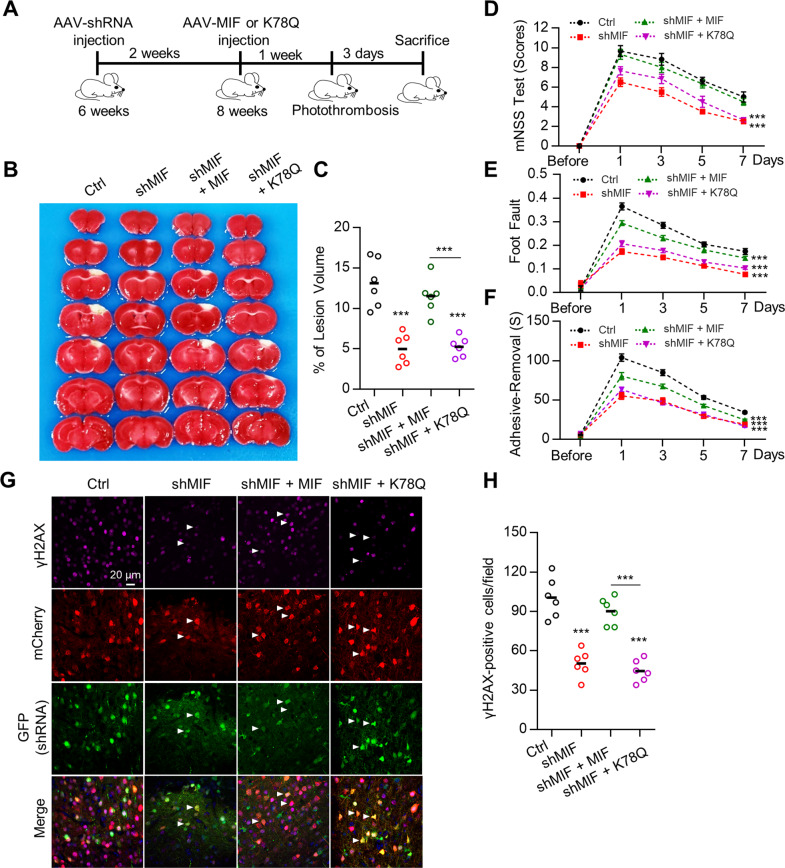
MIF K78 acetylation impaired neuronal death after ischemia. **A** Diagram of the experimental procedure. **B** MIF K78 acetylation attenuated ischemic neuronal death. Mice were injected with AAV-shMIF-GFP together with either AAV-MIF-2A-mCherry or AAV-K78Q-2A-mCherry and ischemia was induced. Infarct volume was analyzed by TTC staining in brain sections. **C** Quantification of the lesion volume is shown in (**B**). Data shown were independent points and mean; one-way ANOVA; *n* = 6; ***p < 0.001. **D**–**F** Neurological deficits were evaluated by the mNSS test (**D**), foot fault assays (**E**), and adhesive-removal test (**F**). Quantification analysis of behavioral tests. Data shown were mean ± SEM; two-way ANOVA; *n* = 6; ***p < 0.001. **G** Reduced DNA fragmentation in neurons expressing MIF K78Q after ischemic stroke. The brain sections infected with indicated AAV were stained with an anti-γH2AX antibody. Arrows indicate AAV-infected neurons. **H** Quantification of the γH2AX-positive cells were shown in (**G**). Data shown were independent points and mean; one-way ANOVA; *n* = 6; ***p < 0.001.

To determine whether MIF acetylation on K78 is involved in mitigating neuronal death in response to ischemia, we used AAV-shMIF to knock down neuronal MIF expression. Two weeks after AAV infection, the MIF protein levels were reduced in cortical neurons (Fig. S5). Two weeks after AAV-shMIF infection, AAVs containing shRNA-resistant WT MIF or acetylation-mimicking MIF K78Q were injected into the cortex. One week later, photothrombotic ischemia was induced in the cortical regions infected with AAV (Fig. 4A). We found that the infarct volume was reduced in the MIF knockdown cortex. Additionally, expression of WT MIF but not the MIF K78Q mutant restored the infarct volume to that observed in WT mice (Fig. 4B, C). We assessed behaviors using the mNSS test, foot fault test, and adhesive-removal test 1, 3, 5, and 7 days after ischemia. Consistent with the infarct data, MIF-deficient mice had better behavioral performance than control mice. MIF-deficient mice expressing WT MIF had behavioral scores equivalent to those of control mice, whereas mice expressing the MIF K78Q mutant were comparable with MIF-deficient mice (Fig. 4D–F), suggesting that control mice and MIF-deficient mice complemented with WT MIF had more severe sensory and motor deficits. DNA fragmentation in cortical neurons was observed in the penumbra region in control mice and MIF-deficient mice complemented with WT MIF after ischemia (Fig. 4G, H). DNA fragmentation was reduced in MIF-deficient mice and MIF-deficient mice expressing K78Q (Fig. 4G, H), suggesting that MIF acetylation attenuates DNA fragmentation in cortical neurons after ischemia. Together, these results indicate that MIF acetylation on K78 protects cortical neurons after ischemia.

### HDAC6 inhibition and aspirin protect cortical neurons by MIF acetylation

We next determined whether MIF acetylation is involved in the protective effect of tubastatin A and aspirin in MNNG-induced cell death. We transfected MIF knockout Neuro2a cells with WT MIF and acetylation-deficient K78R mutant MIF. MIF knockout cells were resistant to MNNG-induced cell death (Fig. S6A, B); however, transfection of MIF knockout cells with WT MIF and the MIF K78R mutant restored MNNG toxicity. Furthermore, consistent with previous results, the HDAC6 inhibitor and aspirin protected cells from MNNG-induced cell death, but transfection of MIF knockout cells with the K78R mutant MIF impaired the protective effects of tubastatin A and aspirin (Fig. S6A, B). These results indicate that tubastatin A and aspirin protect cells from MNNG-induced cell death through MIF K78 acetylation.

**Fig. 5:**
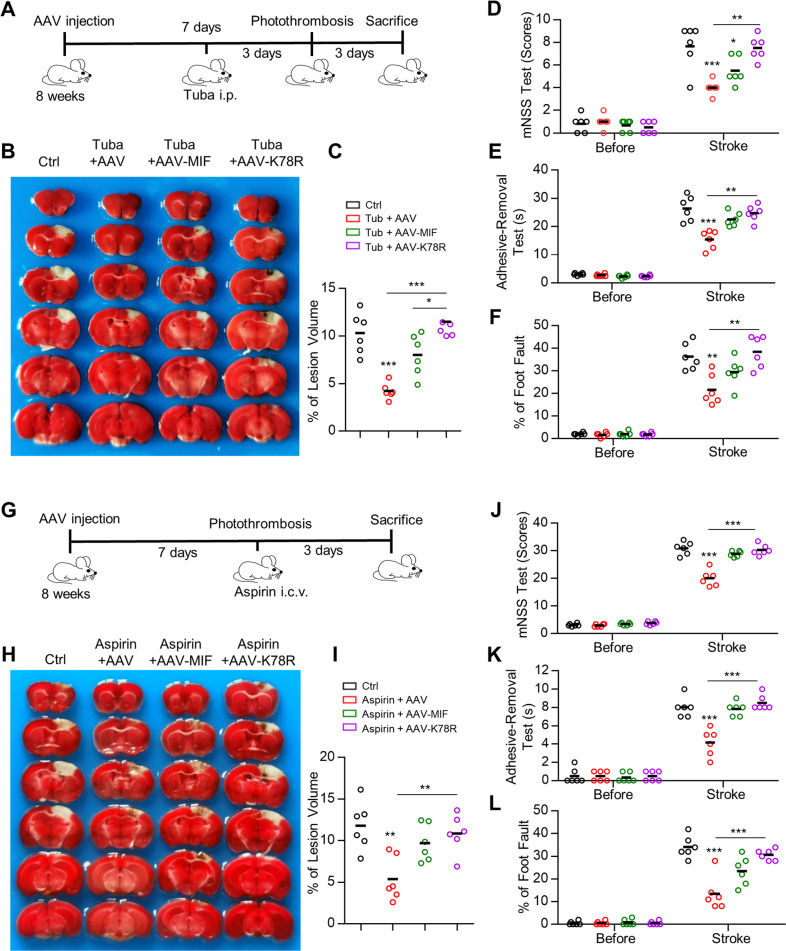
HDAC6 inhibitor and aspirin protect neuronal death through increasing MIF acetylation after ischemia. **A** Diagram of the experimental procedure. **B** HDAC6 inhibitor protects against ischemia-induced neuronal death through MIF acetylation. Mice were infected with either AAV-MIF-2A-mCherry or AAV-K78R-2A-mCherry and administered tubastatin A every day for 3 days. Ischemia was then induced and the infarct volume was analyzed by TTC staining in brain sections. **C** Quantification analysis of the lesion volume is shown in (**B**). Data shown were independent points and mean; one-way ANOVA, *n* = 6; *p < 0.05; ***p < 0.001. **D**–**F** Neurological deficits were evaluated by the mNSS test (**D**), adhesive-removal test (**E**), and foot fault assays (**F**). Data shown were independent points and mean; two-way ANOVA; *n* = 6; *p < 0.05; **p < 0.01; ***p < 0.001. **G** Diagram of the experimental procedure. **H** Aspirin protects against ischemia-induced neuronal death through MIF acetylation. Mice were infected with AAVs and administered aspirin. Ischemia was induced and infarct volume was analyzed by TTC staining in brain sections. **I** Quantification analysis of the lesion volume is shown in (**H**). Data shown were independent points and mean; one-way ANOVA, *n* = 6; **p < 0.01. **J**–**L** Neurological deficits were evaluated by the mNSS test (**J**), adhesive-removal test (**K**), and foot fault assays (**L**). Data shown were independent points and mean; two-way ANOVA; *n* = 6; ***p < 0.001.

To determine whether MIF acetylation is involved in the protective effects of HDAC6 inhibitors in vivo, we injected AAV containing WT MIF or MIF K78R mutant into the cortex. Tubastatin A was administered for 3 days, and then ischemia was induced (Fig. 5A). As shown in Fig. 5B, C, the infarct volume was significantly reduced in the cortex of tubastatin A-treated mice 3 days after ischemia. However, tubastatin A-treated mice expressing the MIF K78R mutant restored the infarct volume to the levels observed in control mice (Fig. 5B, C). Consistent with the infarct data, tubastatin A-treated mice had better behavior scores than control mice, while tubastatin A-treated mice expressing the MIF K78R mutant had behavior scores equivalent to those of control mice (Fig. 5D–F).

To determine whether aspirin can protect neurons from ischemic stroke through MIF acetylation, AAV containing WT MIF or MIF K78R mutant was injected into the cortex. One week after infection, aspirin (200 mM, 2 μl) was injected into the lateral ventricle, followed by an ischemic stroke model (Fig. 5G). Consistent with HDAC6 inhibition, the infarct volume was decreased in the aspirin-treated cortex 3 days after ischemia (Fig. 5H, I). In contrast, aspirin treatment of the cortex expressing the MIF K78R mutant restored the infarct volume to the levels in control mice (Fig. 5H, I). The mice administered aspirin had better behavior scores than control mice, while aspirin-treated mice expressing the MIF K78R mutant had behavior scores equivalent to those of control mice (Fig. 5J–L). Together, these results indicate that HDAC6 inhibition or aspirin protects neurons from ischemia through MIF acetylation.

### MIF K78Q mutant mice displayed attenuated brain injury after ischemia

**Fig. 6:**
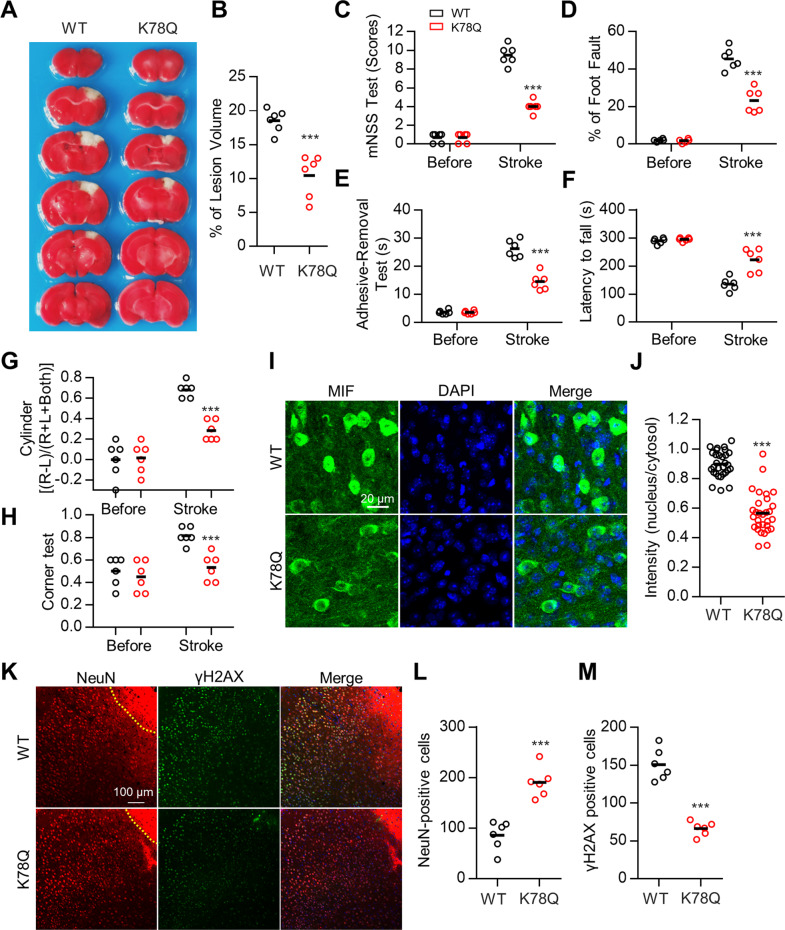
Reduced brain injury in MIF K78Q knock-in mice after ischemia. **A** Attenuated infarct volume in MIF K78Q mice after ischemic stroke. Ischemia was induced in WT and MIF K78Q mice and infarct volume was analyzed by TTC staining in brain sections. **B** Quantification analysis of lesion volume shown in (**A**). Data shown were independent points and mean; unpaired *t*-test; *n* = 6; ***p < 0.001. **C**–**H** Quantitative analysis of behavioral tests. Neurological deficits were evaluated by the mNSS test (**C**), foot fault assays (**D**), adhesive-removal test (**E**), rotarod assays (**F**), cylinder test (**G**), and corner test (**H**). Data shown were independent points and mean; two-way ANOVA; *n* = 6; **p < 0.01; ***p < 0.001. **I**–**M** Reduced MIF protein translocation to the nuclei of cortical neurons in MIF K78Q mice after ischemia. Ischemia was induced in WT and MIF K78Q mice. Brain sections were stained with anti-MIF antibody (**I**), anti-NeuN, and anti-γH2AX antibodies (**K**). **J** Quantification analysis of MIF intensity in nucleus/cytosol shown in (**I**). Data shown were mean ± SEM; unpaired *t*-test; *n* = 13 cells from three mice; ***p < 0.001. **L**, **M** Quantification analysis of NeuN-positive cells (**L**) and γH2AX-positive cells (**M**) shown in (**K**). Data shown were independent points and mean; unpaired *t*-test; *n* = 6 sections from three mice; ***p < 0.001.

Finally, we generated MIF K78Q mutant mice to study MIF K78 acetylation in neuroprotection after ischemia. The MIF protein level in the cortex was comparable between WT and K78Q mutant mice (Fig. S7A, B). The K78Q mice appeared normal, and no significant difference in brain structure was observed in K78Q mutant mice compared with WT littermates (data not shown). We next induced ischemia in the WT and K78Q mice and found that the infarct volume was reduced in the K78Q mutant cortex on Day 3 after ischemia (Fig. 6A, B). Consistent with the infarct data, the K78Q mutant mice had better performance in the behavioral tests than WT littermates (Fig. 6C–H). MIF protein was localized in the nuclei of cortical neurons in the penumbra region of WT mice; in contrast, MIF K78Q protein was primarily observed in the cytosol of cortical neurons after ischemia (Fig. 6I, J), suggesting that acetylation on K78 attenuates MIF translocation into the nucleus after ischemia. The number of NeuN-positive cortical neurons was significantly increased in the penumbra region of K78Q mice compared with WT mice (Fig. 6K, L), and DNA fragmentation in cortical neurons in the penumbra region was reduced in the K78Q mutant mice (Fig. 6K, M). Additionally, the number of GFAP-positive and Iba1-positive cells was reduced in the K78Q mutant cortex compared with the WT cortex (Fig. S6C–F). Together, these data are consistent with the results described above and support the model of MIF K78 acetylation attenuating neuronal death after ischemia by preventing translocation of MIF to the nucleus, which would otherwise cause DNA fragmentation.

## Discussion

**Fig. 7:**
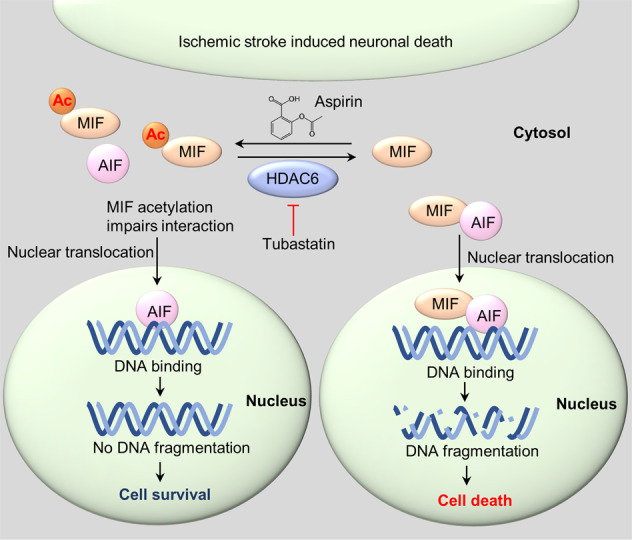
Schematic model demonstrating the mechanism by which MIF K78 acetylation protects neurons from ischemic injury.

Previous studies have shown that inhibition of HDAC6 protects neurons in many neurological disorder models, including Alzheimer’s disease [12], Parkinson’s disease [13, 39], Huntington’s disease [14], amyotrophic lateral sclerosis (ALS) [15], Charcot-Marie-Tooth disease [16, 17], and ischemic stroke [18]. Several proteins have been identified as substrates of HDAC6, including α-tubulin, cortactin, Hsp90 and peroxiredoxin [8–11]. Acetylation of these proteins regulated by HDAC6 may modulate protein functions and contribute to neurological disorders. For example, acetylation of α-tubulin in neurons is impaired in many neurodegenerative disorders, which may attenuate microtubule stability and axonal transport in neurons [14, 16, 17]. HDAC6-mediated Hsp90 deacetylation regulates the chaperone activity of Hsp90, which may be implicated in protein aggregation in neurodegenerative disorders [10, 40]. Peroxiredoxins, which catalyze H_2_O_2_ reduction, are elevated in various neurodegenerative disorders [41]. Acetylation of peroxiredoxins increases the reducing activity, which may regulate redox status in neurological diseases [11, 42, 43]. In this study, we found that MIF K78 acetylation was upregulated by HDAC6 inhibitor or aspirin treatment. Mechanistically, MIF K78 acetylation impaired the interaction between MIF and AIF, which reduced MIF translocation into the nucleus (Fig. 3). Expression of the acetylation-mimicking MIF K78Q mutant suppresses ischemia-induced DNA fragmentation and neuronal death. This was true in both WT mice with AAV-infected cortex expressing the mutant gene and in MIF K78Q mice (Figs. 4, 6). Moreover, the MIF K78R mutant reversed the protective effect of HDAC6 inhibition and aspirin treatment in response to ischemic stroke (Fig. 5). After ischemic stroke, both *Hdac6* mRNA and HDAC6 protein levels were increased in the cortex (Fig. S1E, G). Increased HDAC6 led to reduced MIF K78 acetylation in neurons within the ischemic cortex (Fig. 2L, M). Attenuated MIF K78 acetylation promotes MIF binding to AIF, followed by MIF nuclear translocation and neuronal death. Moreover, ischemic neurons treated with an HDAC6 inhibitor or aspirin showed increased MIF acetylation and reduced nuclear-localized MIF. Reduced nuclear MIF attenuates ischemia-induced DNA fragmentation and neuronal death (Fig. 7). Thus, high MIF K78 acetylation may contribute to neuronal protection after ischemic stroke and potentially other neurodegenerative diseases.

In this study, we identified HDAC6 as the deacetylase of MIF based on the following evidence. First, acetylated MIF was identified by mass spectrometry in the HDAC6-deficient cortex (Table S1). Second, MIF acetylation was dramatically increased in cells treated with the pan-HDAC inhibitor trichostatin A (TSA) or the HDAC6 inhibitor tubastatin A (Fig. 1A, B). Third, overexpression of HDAC6 suppressed MIF acetylation, and the second deacetylase domain was required for MIF deacetylation (Fig. 1I, J). Fourth, MIF acetylation was increased in the brain lysate of HDAC6-deficient mice (Fig. 2J, K). Finally, MIF K78 was revealed as the major acetylation site in the biochemical and mass spectrum results (Figs. 1K, 2E). These results demonstrate that HDAC6 deacetylates MIF. Importantly, MIF K78Q mice, which mimic the biochemical effects of K78-acetylated MIF, displayed reduced infarct brain injury and improved behavioral deficits after ischemic stroke, indicating that MIF K78 acetylation has neuroprotective effects.

Aspirin is widely used for the prevention of primary and especially secondary cardiovascular events [44]. Additionally, aspirin can reduce inflammatory reactions and lower the risk of developing solid cancers [45]. Aspirin treatment in the brain slice has been shown to protect neurons from excitatory toxicity. However, neuroprotective effects have not been observed in clinical studies, possibly due to poor blood–brain barrier penetration and low local concentration in the brain. The effective concentration of aspirin in the brain slice was 3 mM [33], which is much higher than the concentration (0.3 mM) in the mouse brain by oral administration [46]. In this study, aspirin, which was directly injected into the lateral ventricle, protected neurons in ischemic stroke (Fig. 5G–K). Therefore, efficient delivery of aspirin to the injured regions without affecting blood vessels could be beneficial for ischemic patients.

MIF has been implicated in several biological functions, including chemo-attraction, cytokine, and chaperone-like activity [47]. MIF was one of the first cytokines discovered to regulate inflammatory responses [48]. Extracellular MIF can protect the heart during ischemia-reperfusion [49]. In the ALS mouse model, the chaperone activity of MIF has been demonstrated to prevent misfolded SOD1 accumulation in mitochondria, and increasing MIF expression rescues mutant SOD1-induced neuronal death [50, 51]. Disruption of MIF in mice results in an enlarged infarct area when ischemia is modeled using the middle cerebral artery ligation (MCAI) method [52]. In contrast, MIF-deficient mice showed a smaller infarct volume when ischemia was modeled using the transient middle cerebral artery occlusion (tMCAo) method [53] and the middle cerebral artery occlusion (MCAo) method [25]. Consistent with these studies, our results revealed reduced infarct volume in the MIF-deficient cortex infected with AAV-shMIF. Furthermore, infarct volume and neuronal death were reduced in MIF K78Q mutant mice after ischemia was induced. Together, our results suggest that MIF K78 acetylation plays an important role in neuroprotection in the ischemic cortex.

## Materials and methods

### Reagents and antibodies

Chemicals were purchased from Sigma–Aldrich Company (St. Louis, MO, USA) unless otherwise indicated. The HDAC inhibitor trichostatin A (TSA) (ab120850) and HDAC6 inhibitor tubastatin A (ab141415) were purchased from Abcam (Cambridge, MA, USA). The primary antibodies used were rabbit anti-HDAC6 (cat. 7558, Cell Signaling (Danvers, MA, USA), 1:1000 for WB, 1:200 for staining), rabbit anti-GAPDH (cat. 5174, Cell Signaling, 1:10000 for WB), rabbit anti-Ac-K (cat. 9441, Cell Signaling, 1:1000 for WB), rabbit anti-Myc (cat. PA9064, Abmart, 1:1000 for WB), rabbit anti-Flag (cat. F7425, Sigma, 1:2000 for WB), mouse anti-NeuN (cat. MO22122, Neuromics (Edina, MN, USA), 1:1000 for staining), rabbit anti-GFAP (cat. Z0334, Dako (Kyoto, Japan), 1:1000 for staining), rabbit anti-Iba1 (cat. Ab5076, Abcam, 1:500 for staining), rabbit anti-γH2AX (cat. 9718, Cell Signaling, 1:1000 for WB, 1:500 for staining), and MIF (cat. Ab187064, Abcam, 1:1000 for WB, 1:500 for staining). The MIF K78 acetylation antibody was generated by ABclonal Biotechnology (Shanghai, China).

### Cell culture and DNA transfection

HEK293T cells and Neuro2a cells were cultured in Dulbecco’s modified Eagle’s medium (DMEM) supplemented with 10% fetal bovine serum (FBS) in an incubator in a humidified atmosphere containing 5% CO_2_ at 37 °C. Cells cultured at approximately 70% confluence were transfected with plasmid DNA using Lipofectamine 2000 (cat. 11668, Invitrogen) following the manufacturer’s instructions.

MIF knockout cells were generated as previously described [54]. Briefly, Neuro2a cells were transfected with the PX330-GFP plasmid. GFP-positive cells were sorted by FACS and placed into 96-well plates, and each well was seeded with a single cell. After culturing for 10 days, cells from individual wells were subcultured into 35 mm dishes. The genotypes of MIF knockout cells were verified by PCR sequencing and immunoblotting.

Cell death induced by methylnitronitrosoguanidine (MNNG) (Sigma) in Neuro2a cells was performed as previously described [25]. Briefly, Neuro2a cells with the indicated genotypes were transfected with the indicated constructs. Two days after transfection, the Neuro2a cells were exposed to MNNG (50 μM) for 15 min. The Neuro2a cells were stained with propidium iodide and Hoechst 33342 12 h after MNNG treatment to determine cell death.

### Animals

All mice used in the experiments were male and 2–3 months of age, and they weighed 24–30 g. The mice were housed under controlled temperature, humidity, and light conditions (12 h light/dark cycle) with water and rodent chow diet freely available. The HDAC6 mutant mice were gifts from Pro. Yao TP (Duke University) [55]. MIF K78Q mice were generated using homologous recombination by Shanghai Model Organisms Center, Inc. Genotypes were confirmed by Sanger sequencing or PCR analysis of tail DNA.

The K78Q mouse genotyping primers were as follows: forward: 5′-ATTGGGGTGTGATGGCAGG-3′ and reverse: 5′-TGGGAAGGAAAATGGGGC-3′. The WT band was ~278 bp, and the mutant band was ~278 bp. The HDAC6 mutant mouse genotyping primers were as follows: forward: 5′-CTGGTTCGTCTGAAGACA-3′, reverse: 5′-GTGGACCAGTTAGAAGCC-3′ for the WT allele and forward: 5′-CCATGACCGAGATCGGCGAGCA-3′, reverse: 5′-CGTGAATTCCGATCATATTCAAT-3′ for the mutant allele.

All mouse experiments were conducted in accordance with the guidelines of the Institutional Animal Care and Use Committee (IACUC) and under an approved IACUC protocol of Xuzhou Medical University (IACUC 20140404W009).

### Photothrombotic ischemia

The cortical injury was induced by photothrombotic ischemia as previously described [56, 57]. Briefly, mice were anesthetized with ketamine (100 mg/kg) and xylazine (10 mg/kg) and then injected with 1% rose bengal (i.p., 100 mg/kg, Sigma) dissolved in 0.9% saline. Ten minutes later, a large part of the sensorimotor cortex centered approximately 2 mm lateral to the bregma was exposed to the cold light for 15 min. The control mice received the same operation without rose bengal injection. After the operation, the mice were placed on a heating pad to maintain body temperature, and 0.9% saline was given by subcutaneous injection.

### Drug administration

The HDAC6 inhibitor tubastatin A (10 mg/kg) was administered to WT mice by intraperitoneal injection (i.p.) every day for 3 days. The photothrombotic ischemia method was then implemented. After photothrombosis, tubastatin A was administered for another 3 days. Aspirin (200 mM, 2 μl) was injected into the lateral ventricle (reference to bregma: anteroposterior = −0.8 mm, lateral = −1.0 mm, depth = 2.5 mm) before ischemia. Then, photothrombotic ischemia was performed in the ipsilateral cortex, and behavioral tests were performed 3 days after photothrombosis.

### Adeno-associated virus-9 (AAV-9) preparation

The short hairpin interfering RNA constructs were generated by BrainVTA Co., Ltd. (Wuhan, China) in the pAAV2/9-H1-shRNA-CAG-eGFP backbone. The MIF shRNA (targeting MIF mRNA) sequence was 5′-GGGTCTACATCAACTATTA-3′. The PCR-amplified mouse MIF (NM_010798.2) was cloned into the rAAV-hsyn-2a-mCherry-WPRE-pA AAV-2/9 vector to generate MIF expression constructs. Adeno-associated virus-9 was packaged by BrainVTA Co., Ltd. (Wuhan, China).

### Virus Injection

The mice were anesthetized and fixed on a stereotaxic plate (RWD Life Science, Shenzhen, China), and three different regions surrounding the ischemic injury were selected for drilling by a hand drill (RWD). One microliter of AAV (containing 1 × 10^13^ particles) was slowly injected into the brain cortex (DV = 1.5 MM, 10 min), and 10 min later, the needle was withdrawn and changed to another region. After all three regions were injected with AAV, the holes were sealed, and the wound was sutured. After 4 weeks, the majority of neurons were infected with AAV, and photothrombosis was conducted to produce an ischemia model.

### Behavioral tests

All animals were trained two times a day for 5 days before photothrombotic ischemia surgery to reduce anxiety and ensure that the baseline levels of the control and experimental groups were the same.

A modified neurological severity score (mNSS) evaluation, adhesive-removal test, and foot fault test were performed before ischemia and then 1, 3, and 7 days after ischemia. The mNSS is a composite of motor, sensory, balance, and reflex tests. It includes motor testing, such as raising the mouse by its tail to assess forelimb flexion, hindlimb flexion, and head movement; the ability to walk straight when placed on the ground; a timed beam balance test; sensory (visual and tactile) tests; test of reflexes to sudden auditory stimuli; and corneal reflex. Neurological function was graded on a scale of 0 to 14 (normal score = 0; maximal deficit score = 14), with one point awarded for the exhibition of specific abnormal behavior or for lack of a tested reflex [58, 59].

The adhesive-removal test is a sensitive method for assessing sensorimotor deficits in mice [60]. The animal was gently removed from the testing box, and adhesive dots (4 mm in diameter) were attached to the forepaws. Then, the animal was returned to the box, and it started to remove the adhesive dots with its teeth. The procedure was repeated twice, and the mean time taken to successfully remove the dots from each paw was recorded. The time required to remove the dots was used to determine sensorimotor performance.

The corner test was conducted as previously described [61]. The mice were allowed to proceed to a corner (30° angle) between two cardboard pieces, each with dimensions of 30 × 20 × 1 cm^3^. When the mice entered the deep part of the corner, they reared forward and upward and then turned back to exit the corner. They could turn to the left or the right, and the direction of turning was recorded. Twenty trials were performed for each mouse, and the percentage of right turns was calculated.

The cylinder test was designed to evaluate locomotor asymmetry and performed as previously described [61]. The animal was placed in a transparent cylinder (9 cm in diameter and 15 cm in height). When it moved, its forelimb reared against the cylinder wall. The use of the forelimb was defined by the placement of the whole palm on the wall of the cylinder to support its body. When one forelimb contacted the wall to the rear, one forelimb (nonimpaired or impaired movement in accordance with the actual situation) was recorded. The simultaneous use of both forelimbs to contact the wall of the cylinder during a full rear was recorded as both movements. When one forelimb (for example, the impaired forelimb) contacted the wall to the rear and then the nonimpaired forelimb contacted the wall but the impaired forelimb remained on the wall, both movements were recorded, including impaired forelimb movement. When the mouse explored the wall laterally, alternating its two forelimbs, the action was recorded as two movements. A total of 20 movements were recorded for 10 min. The final score was calculated as follows:

(nonimpaired forelimb movement – impaired forelimb movement)/(nonimpaired forelimb movement + impaired forelimb movement + both movements)

The rotarod test was employed to evaluate the motor coordination and antifatigue ability of mice before and after photothrombotic ischemia treatment. The mice were trained on the accelerating rotor mode (speeds from 4 to 40 rpm/min for 5 min) three times a day with a 10-min rest each time. The mice were trained for 5 days, and the mice staying on the rotating rod for more than 300 s were selected to generate the stroke model. The times each mouse spent on the rotating rod instrument before photothrombotic ischemia and 3 days after ischemia were recorded.

2,3,5-Triphenyl tetrazolium chloride (TTC) staining

Animals in different groups were sacrificed after ischemic stroke for brain infarct formation evaluation. The fresh brains were removed and kept at −20 °C for 30 min and then rapidly sliced into 1 mm coronal sections. Then, the sections were incubated in 2% 2,3,5-triphenyl tetrazolium chloride (TTC) in phosphate buffer and stained for 20 min at 37 °C in the dark. The sections were fixed with 4% paraformaldehyde (PFA) at 4 °C overnight. The sections were selected and scanned with a camera. The infarct areas were measured using ImageJ software. The total infarct volume was calculated with the following formula: (contralateral hemisphere volume – noninfarcted ipsilateral hemisphere volume)/contralateral hemisphere volume × 100%.

### Mass spectrum analysis

The identification of acetylated proteins and MIF acetylation were performed as previously described [38]. In brief, cortex samples from HDAC6 mutant mice were incubated with 5 mM dithiothreitol (DTT), alkylated with 10 mM iodoacetamide, and digested in 2 µg trypsin (Promega, Madison, WI, USA) in 1 M urea and 50 mM NH_4_HCO_3_ at 37 °C overnight. Digested peptides were desalted using an SPE C18 desalination column (Waters WAT051910). The desalted samples were immunoprecipitated with Anti-Ac-K antibody beads (Cell Signaling) in IAP Buffer. The enriched acetylated peptides were analyzed on a Q-Exactive HF-X (Thermo Fisher Scientific). The raw MS data acquired were imported into MaxQuant for protein and peptide identification. The MS/MS spectra were run against the database XXXniport_mouse_78986_20151130.fasta.

To identify MIF acetylation site(s), HEK293T cells transfected with Flag-MIF were treated with aspirin and lysed in lysis buffer comprising 50 mM Tris-HCl (pH 7.4), 150 mM NaCl, 0.5% sodium deoxycholate, 1% Triton X-100, 1 mM phenylmethylsulfonyl fluoride (PMSF), 1 mM EDTA, 5 mM sodium fluoride, 2 mM sodium orthovanadate, and protease inhibitors. Cell lysates were immunoprecipitated with an anti-Flag antibody, and the resulting complex was digested in 2 µg trypsin (Promega) in 1 M urea and 50 mM NH4HCO_3_ at 37 °C overnight. Digested peptides were desalted using the MonoSpin C18 desalination column (GL Sciences) and then analyzed on a Q-Exactive HF-X (Thermo Fisher Scientific). The raw MS data acquired were imported into MaxQuant version 1.5.4.1 for protein and peptide identification. The MS/MS spectra were run against the human UniProt FASTA database (release 2016_07, 48863 sequences).

### Bioinformatics analysis

Metascape was used to perform gene ontology (GO) and Kyoto Encyclopedia of Genes and Genomes (KEGG) pathway enrichment analyses based on the top 50 acetylated proteins list of 218 acetylated proteins (https://metascape.org/gp/index.html#/main/step1). Gene set enrichment analysis (GSEA) was performed based on the GO terms associated with gene annotations downloaded from https://bioinf.wehi.edu.au/software/MSigDB/. The top 50 acetylated proteins were uploaded to perform GSEA using R language with the clusterProfiler package. Using the intensity of acetylated proteins to rank the gene list, the enriched pathways were determined. The results were analyzed by a GSEA enrichment plot.

### Immunoblotting

Tissues were lysed/homogenized in lysis buffer (described above in “Mass spectrum analysis”) for 15 min at 4 °C and then centrifuged at 12,000 × *g*. Cell lysates were resolved by SDS–PAGE followed by Western blot analyses using the appropriate antibodies. Primary antibodies were incubated at 4 °C overnight. After washing with TBST buffer, the HRP-conjugated secondary antibodies (Life Technology) were incubated at room temperature for 1 h. Immunoreactive bands were visualized using enhanced chemiluminescence (Pierce), imaged using a Tanon 5200 imaging system, and analyzed with ImageJ (NIH). The band density of target proteins was normalized to the loading control.

### Immunoprecipitation

Tissues or cells were lysed in lysis buffer (described above in “Mass spectrum analysis”) and centrifuged at 12,000 × *g* at 4 °C for 10 min. The supernatant was transferred into a new tube. Supernatants were incubated with the indicated antibodies overnight at 4 °C. The Protein G agarose beads (11243233001, Sigma–Aldrich) were washed twice with 1 mL PBS and centrifuged at 2000 × *g* for 30 s, and the supernatant was removed between washes. Then, the beads and supernatants incubated with the indicated antibody at 4 °C were mixed and gently rocked for 2 h. The agarose beads were washed in lysis buffer three times, and 1x loading buffer was added to the agarose beads to dissociate the immunocomplex from the beads. Immunoblotting was performed with the precipitated proteins.

### Immunostaining

For brain immunofluorescence staining analysis, mice were anesthetized and transcardially perfused with 4% paraformaldehyde (PFA). The brains were dissected, postfixed in 4% PFA at 4 °C for 2 h, and then sectioned into 50-μm-thick slices by a VT1000S vibratome (Leica). The brain sections were pretreated in 0.5% Triton X-100 in phosphate-buffered saline (PBS, pH 7.4) for 1 h, blocked with 10% normal donkey serum, and 0.1% Triton X-100 in PBS for 1 h, and incubated with the indicated primary antibodies at 4 °C overnight. After washing with PBS, the samples were incubated with appropriate secondary antibodies conjugated to Alexa Fluor 488 or Alexa Fluor 546 (1:500, Vector Laboratories (Burlingame, CA, USA)) at room temperature for 1 h. The sections were counterstained with DAPI for 10 min, followed by washing in PBS. Images were acquired by ZEISS LSM880 confocal microscopy.

### Quantification of MIF nuclear translocation

Nuclear translocation of MIF was quantified as described previously [25]. Briefly, immunofluorescent images were acquired by Zeiss LSM880 confocal microscopy with a Z-Stack scan (Range, 20 μm; Slices, 9; Interval, 2.5 μm). The serial images were processed into one image by maximum intensity projection. The fluorescence intensity in the nucleus and cytosol in neurons was measured by ImageJ software, and the nucleus/cytosol ratio was quantified.

### Quantitative PCR analysis

Total RNA was isolated from the cortical cortex by TRIzol extraction (Thermo Fisher Scientific, 15596026) and converted to cDNA using maxima reverse transcriptase (Thermo Fisher Scientific, EP0742). cDNA products were amplified in 20 μl of reaction mixture containing the SYBR Green PCR Kit (Bio-Rad) with the following primers:

*HDAC6* forward: 5′-TGGCCGTATTATTCTTATT-3′, *HDAC6* reverse: 5′-GAGCATTCTTCCTTGTCTT-3′; and *GAPDH* forward: 5′- GCACAGTCAAGGCCGAGAAT-3′.

*GAPDH* reverse: 5′-GCCTTCTCCATGGTGGTGAA-3′. The PCR program comprised an initial step at 95 °C (5 min) followed by 40 cycles of denaturation at 95 °C (15 s) and annealing at 60 °C (1 min). Samples were run in triplicate, and all signals were normalized to the GAPDH intensity. The relative gene expression levels were calculated using the 2^− ∆ ∆Ct^ method.

### Statistical analysis

Statistical analyses were performed using Prism 5 (GraphPad Software). Data were tested for normality by the D’Agostino-Person normality test and homogeneity of variance; no violations of assumptions were found. One-way or two-way analysis of variance (ANOVA) with Tukey’s multiple comparisons post hoc test was used to evaluate the statistical significance of three or more groups of samples, and a two-tailed, unpaired Student’s *t*-test was used for two groups of samples. All tests were two-sided. *p* < 0.05 was considered to be statistically significant.

## Supplementary information

Original Data File

Original Data File

checklist

Figure S1

Figure S2

Figure S3

Figure S4

Figure S5

Figure S6

Figure S7

Supplementary

Supplementary

Supplemetary Table

## Data Availability

All data that support the findings of this study are present in this article and its supplementary materials. Additional data related to this paper may be requested from the corresponding author.
