# Supplementary figures and images for: Macrophage migration inhibitory factor (MIF) acetylation protects neurons from ischemic injury
Source: Cell Death Dis. 2022 May 18;13(5):466. doi: 10.1038/s41419-022-04918-2 (PMC9117661; doi:10.1038/s41419-022-04918-2)

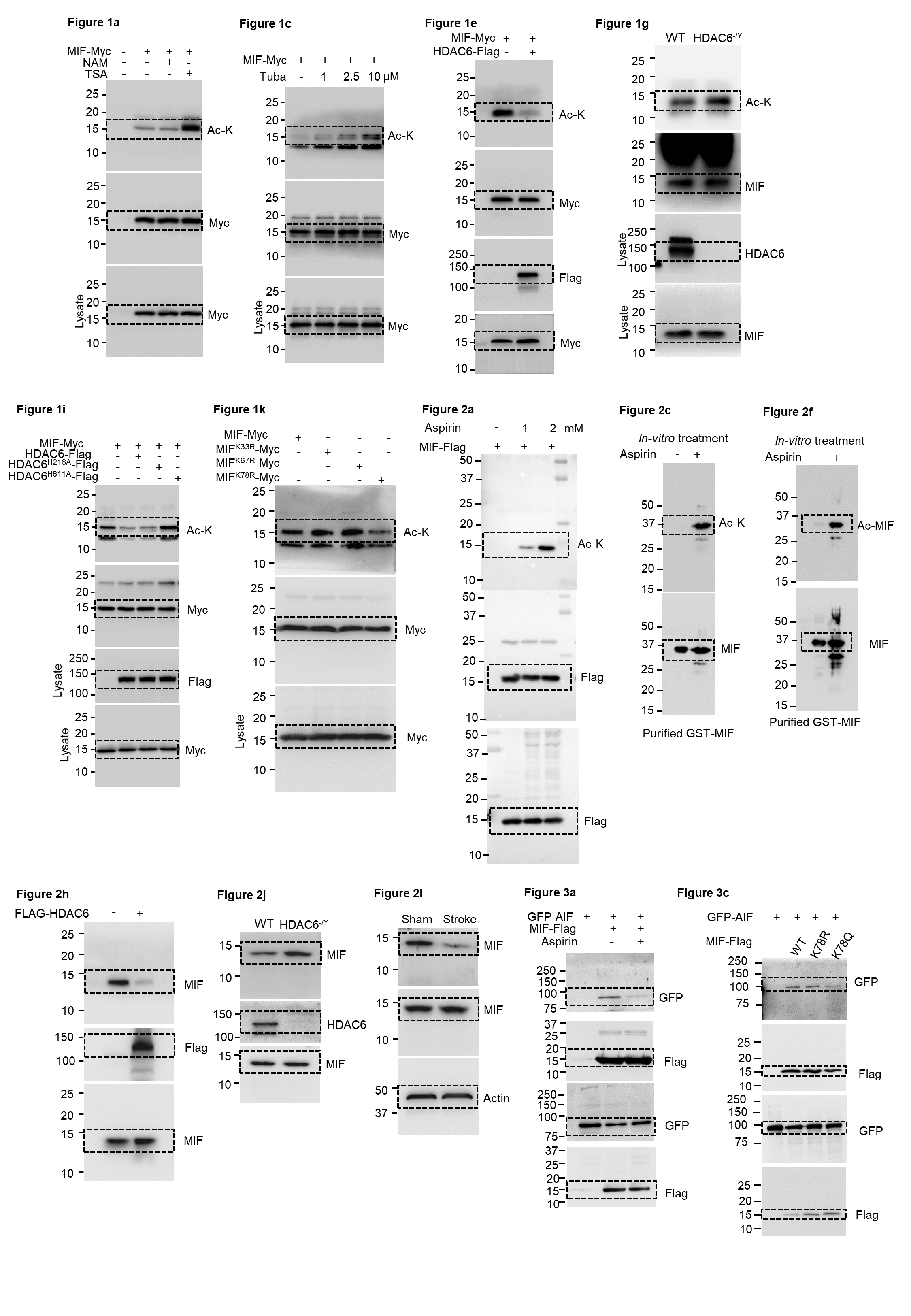

Supplement: Supplementary file 1 — Original Data File [file 41419_2022_4918_MOESM1_ESM.tif]

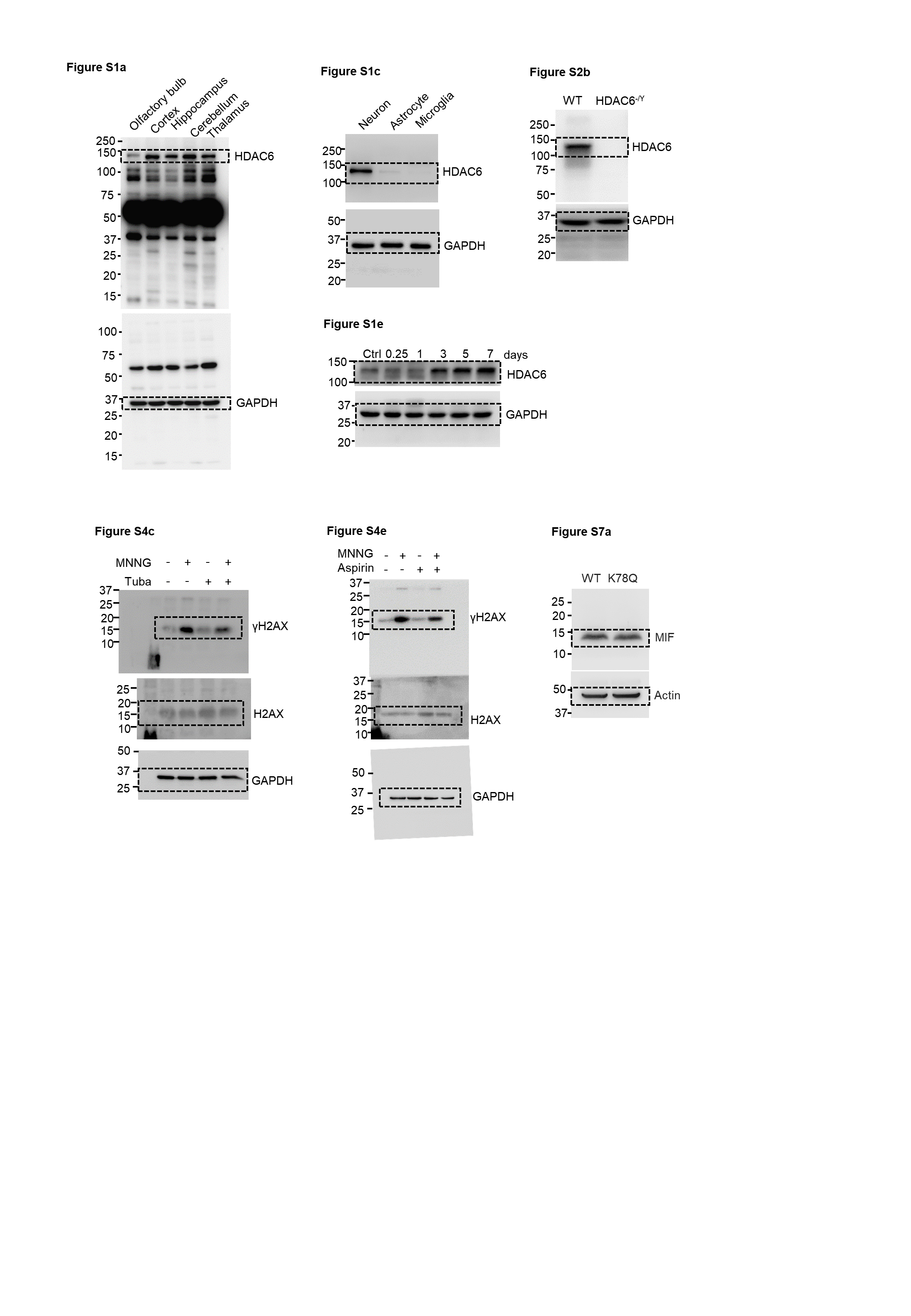

Supplement: Supplementary file 2 — Original Data File [file 41419_2022_4918_MOESM2_ESM.tif]

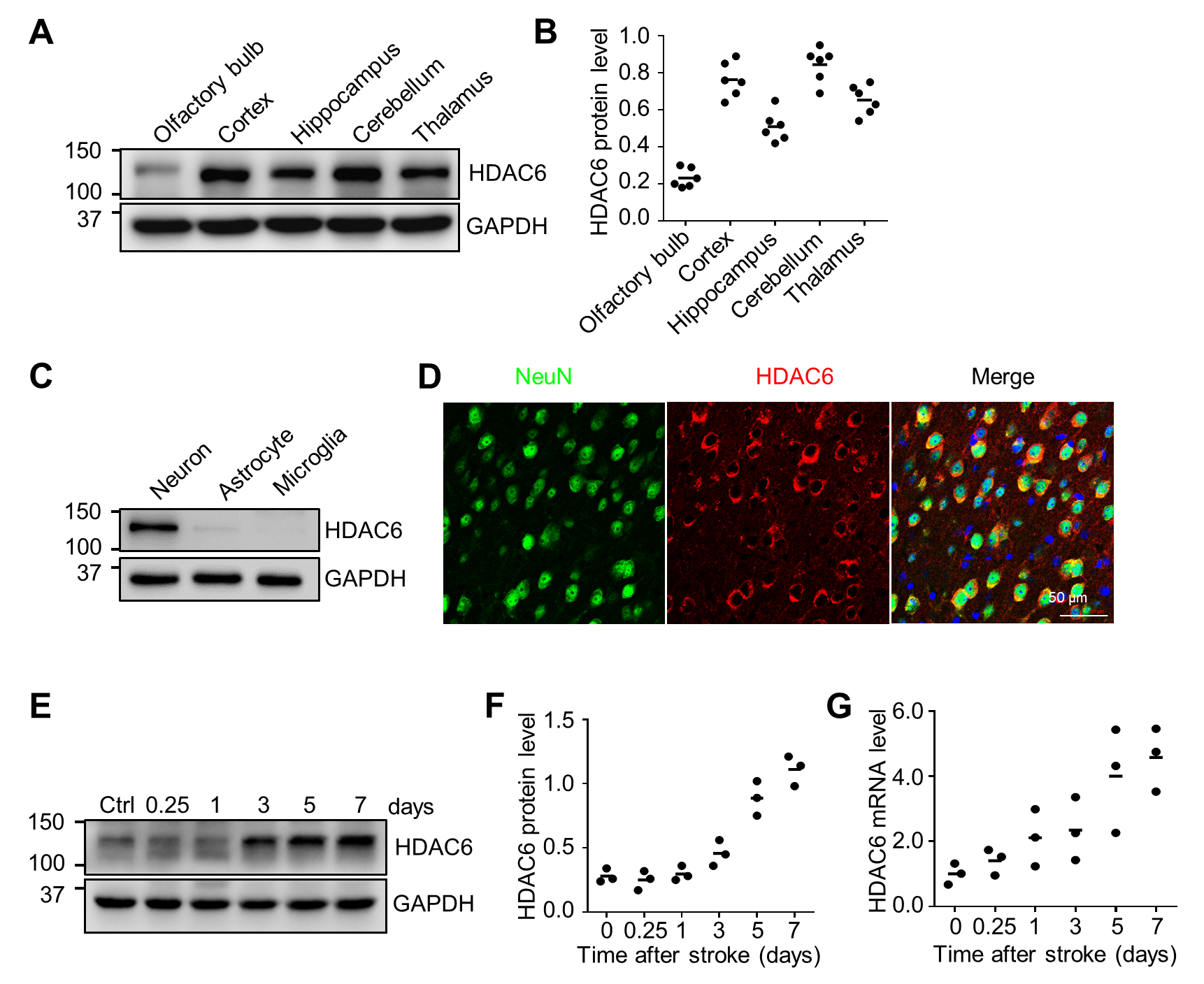

Supplement: Supplementary file 4 — Figure S1 [file 41419_2022_4918_MOESM4_ESM.tif]

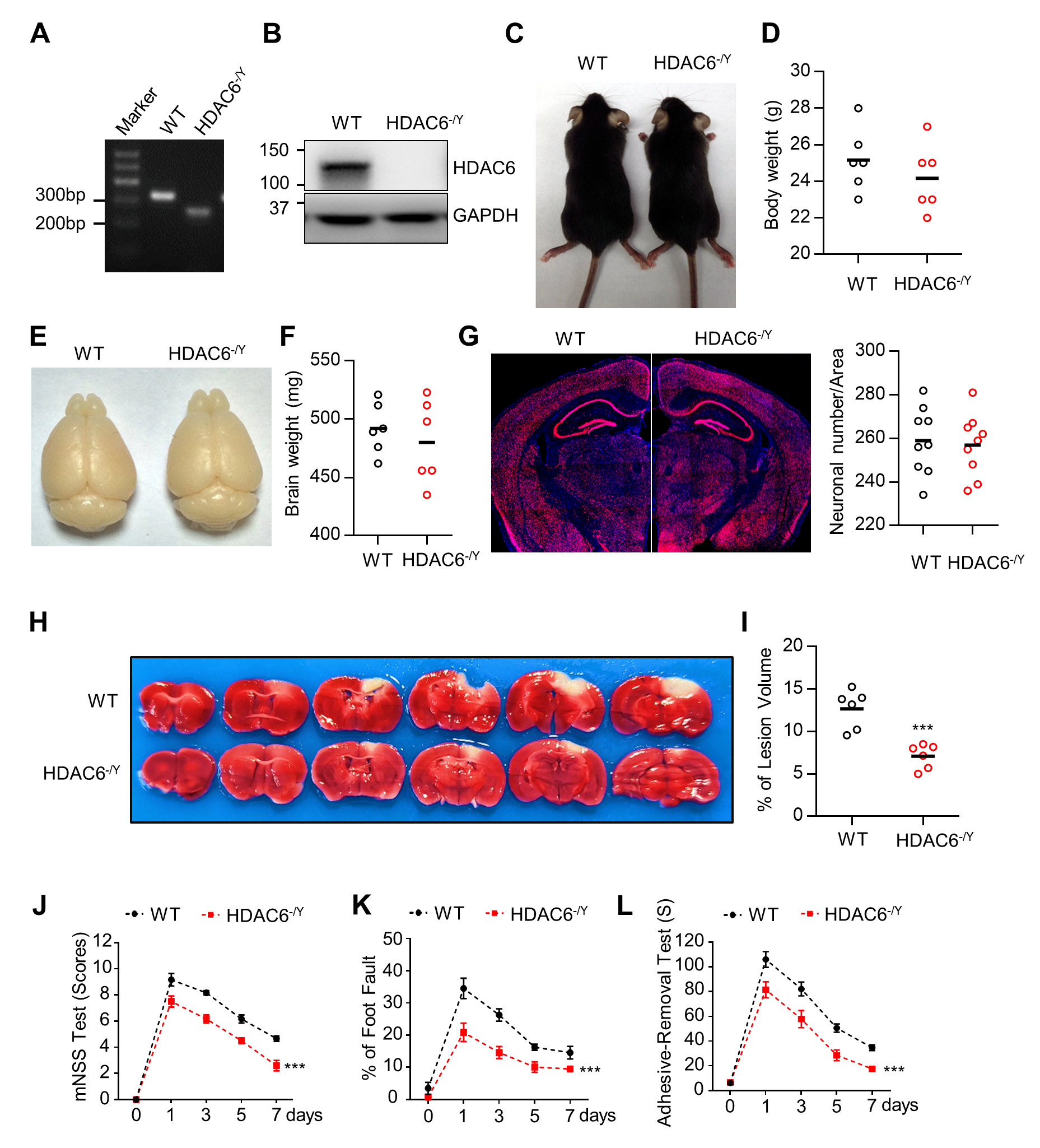

Supplement: Supplementary file 5 — Figure S2 [file 41419_2022_4918_MOESM5_ESM.tif]

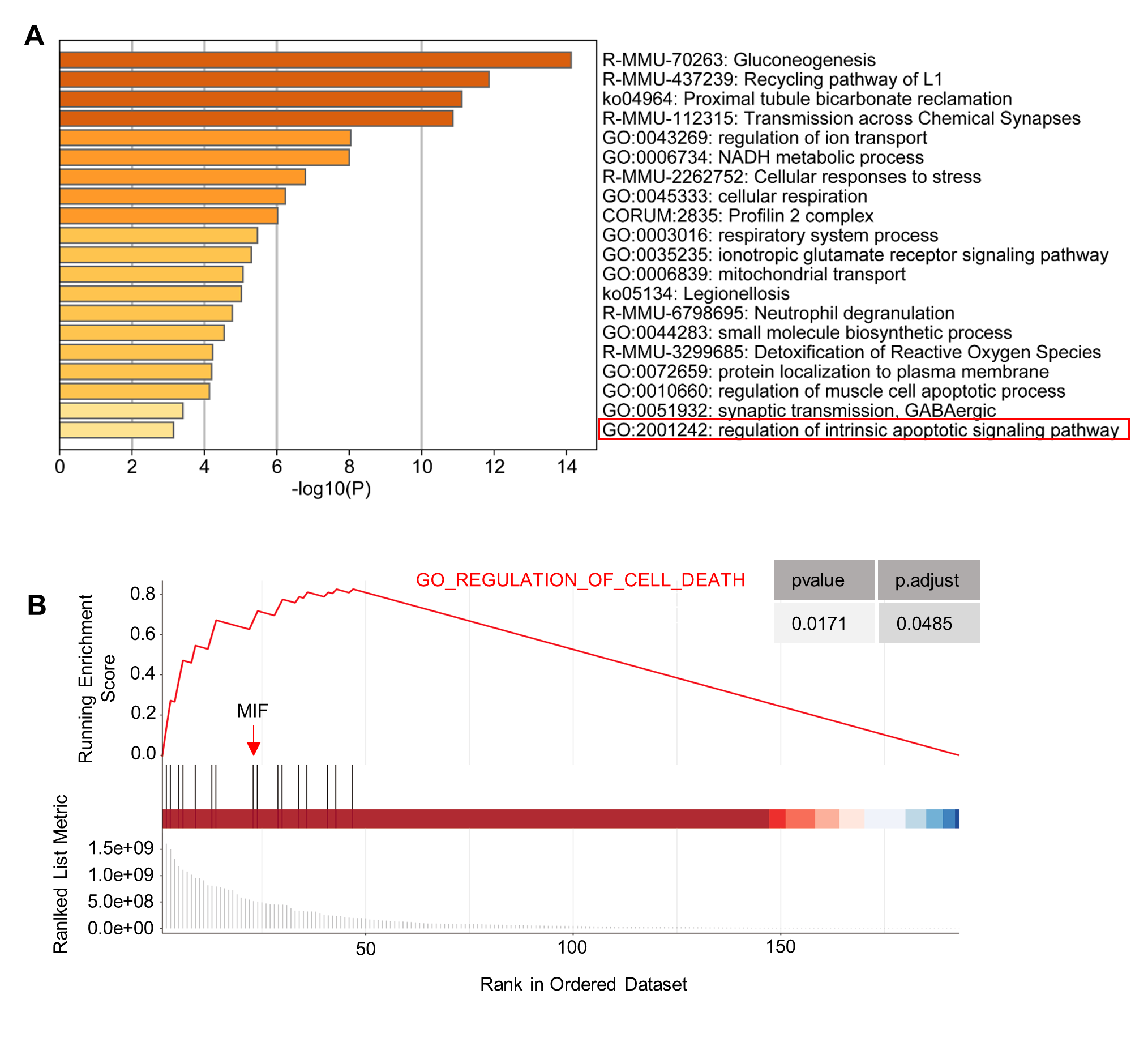

Supplement: Supplementary file 6 — Figure S3 [file 41419_2022_4918_MOESM6_ESM.tif]

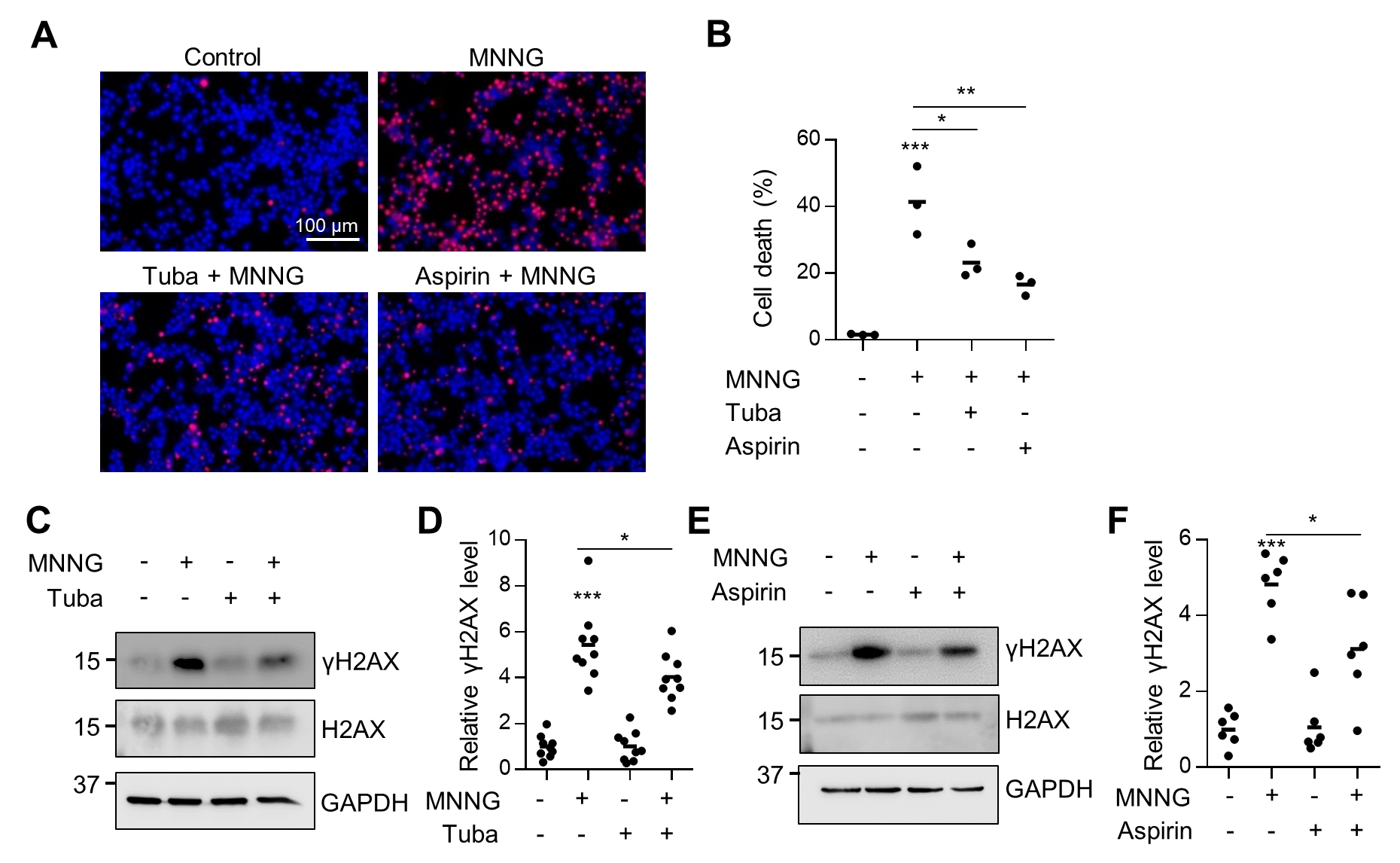

Supplement: Supplementary file 7 — Figure S4 [file 41419_2022_4918_MOESM7_ESM.tif]

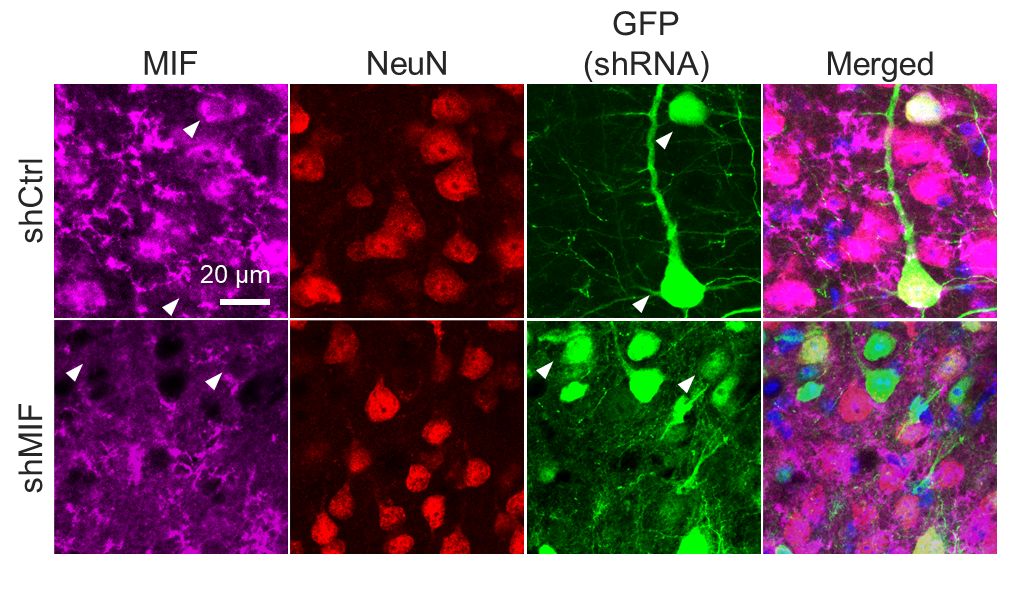

Supplement: Supplementary file 8 — Figure S5 [file 41419_2022_4918_MOESM8_ESM.tif]

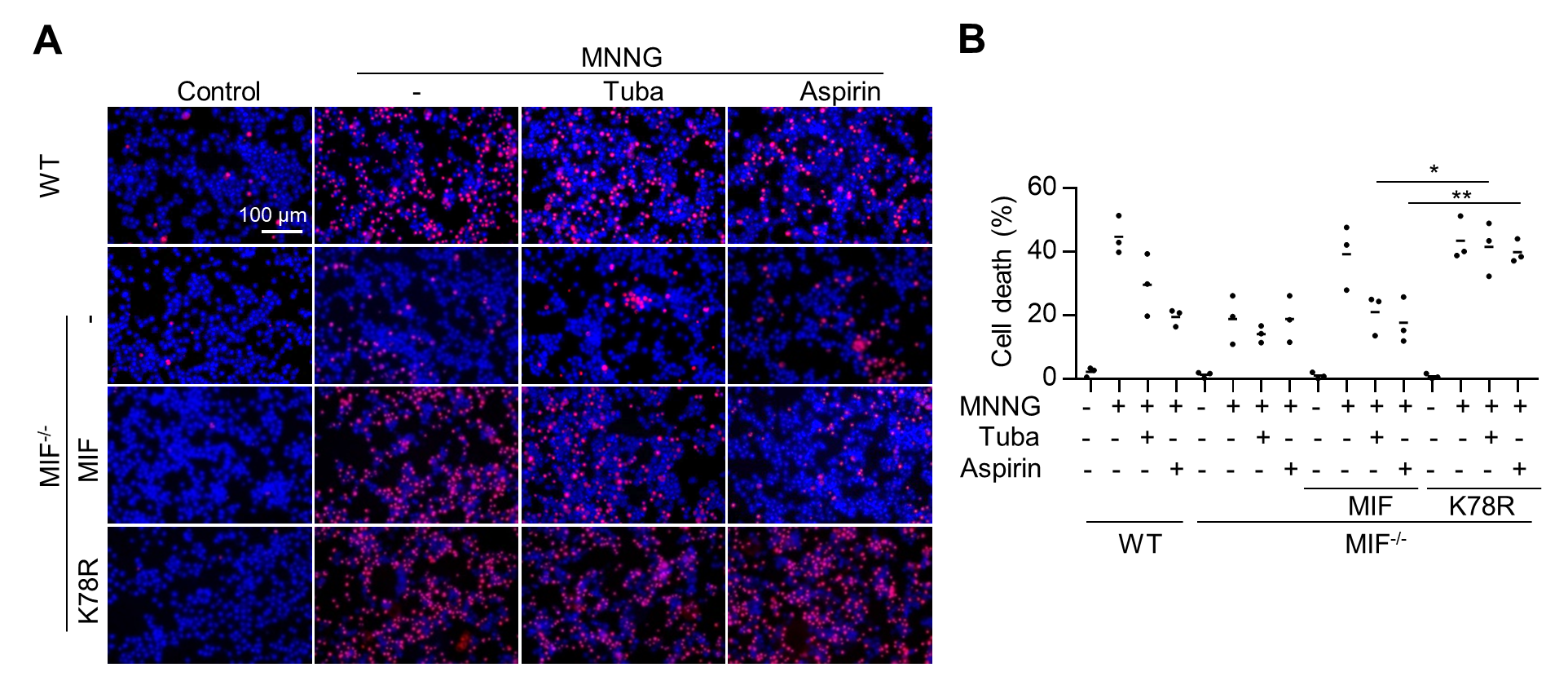

Supplement: Supplementary file 9 — Figure S6 [file 41419_2022_4918_MOESM9_ESM.tif]

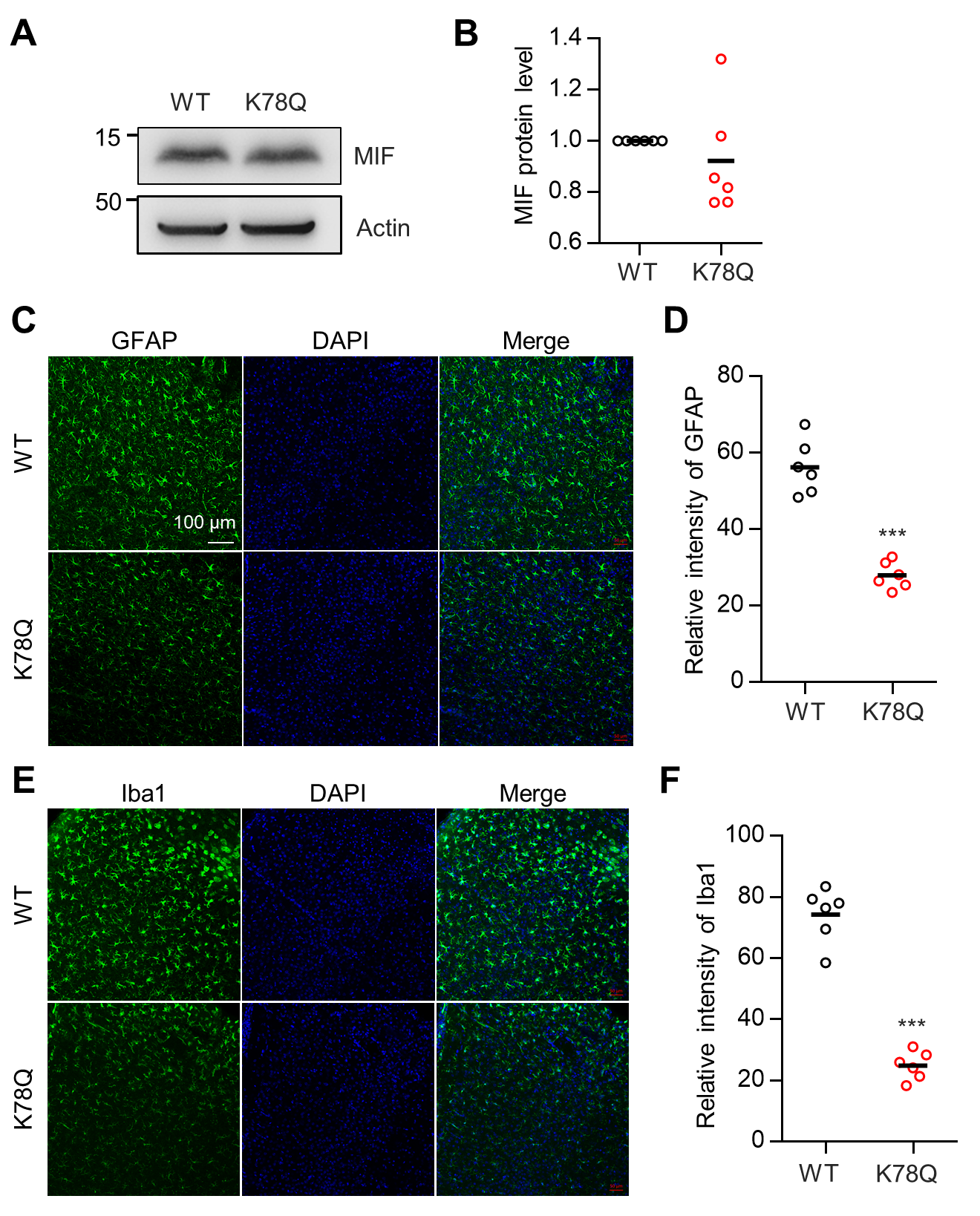

Supplement: Supplementary file 10 — Figure S7 [file 41419_2022_4918_MOESM10_ESM.tif]
