# Supplementary material for: Macrophage migration inhibitory factor (MIF) acetylation protects neurons from ischemic injury
Source: Cell Death Dis. 2022 May 18;13(5):466. doi: 10.1038/s41419-022-04918-2 (PMC9117661; doi:10.1038/s41419-022-04918-2)
Supplement: Supplementary file 11 — Supplementary [file 41419_2022_4918_MOESM11_ESM.pdf]

Editing Certificate

This document certifies that the manuscript

Macrophage migration inhibitory factor (MIF) acetylation protects neurons from ischemic injury

prepared by the authors

Jin-Xia Hu, Wei-Jing Ma, Li-Ying He, Cong-Hui Zhang, Cheng Zhang, Yan Wang, Chao-Nan Chen, Da-Yong Shen, Hui-Min Gao, Rui-Ru Guo, Qian-Qian Ning, Xin-Chun Ye, Gui-Yun Cui, and Lei Li

was edited for proper English language, grammar, punctuation, spelling, and overall style by one or more of the highly qualified native English speaking editors at AJE.

This certificate was issued on **March 25, 2022** and may be verified on the AJE website using the verification code **2D6B-C9DA-FBB7-7703-0F6D**.

Neither the research content nor the authors' intentions were altered in any way during the editing process. Documents receiving this certification should be English-ready for publication; however, the author has the ability to accept or reject our suggestions and changes. To verify the final AJE edited version, please visit our verification page at aje.com/certificate. If you have any questions or concerns about this edited document, please contact AJE at support@aje.com.

AJE provides a range of editing, translation, and manuscript services for researchers and publishers around the world. For more information about our company, services, and partner discounts, please visit aje.com.
