# Supplementary material for: Macrophage migration inhibitory factor (MIF) acetylation protects neurons from ischemic injury
Source: Cell Death Dis. 2022 May 18;13(5):466. doi: 10.1038/s41419-022-04918-2 (PMC9117661; doi:10.1038/s41419-022-04918-2)
Supplement: Supplementary file 12 — Supplementary [file 41419_2022_4918_MOESM12_ESM.docx]

**Supplementary**

**Figure S1. The expression of HDAC6 increased after ischemia.**

(A) Immunoblot analysis of HDAC6 protein levels in different brain regions.

(B) Quantification of relative protein levels of HDAC6 shown in (A) normalized to GAPDH. Data shown are independent points and mean; n = 6.

(C) Immunoblot analysis of HDAC6 protein in primary cultured neurons, astrocytes and microglia.

(D) Brain slices were stained with anti-NeuN antibody (green), anti-HDAC6 antibody (red), and counterstained with DAPI (blue).

(E) Immunoblot analysis of HDAC6 protein levels in the cortex after ischemia. The photothrombotic method was used to induce ischemia in WT mice. The penumbra regions of the cortex were isolated and analyzed by immunoblot on the indicated days after ischemia.

(F) Quantification of relative protein levels of HDAC6 shown in (E) normalized to GAPDH. Data shown are independent points and mean; one-way ANOVA; n = 3; F = 45.510, *p* = 0.007.

(G) qPCR analysis of *Hdac6* mRNA levels in the cortex after ischemia. Data shown are independent points and mean; one-way ANOVA; n = 3; F = 6.623, *p* = 0.004.

**Figure S2. Reduced brain injury in HDAC6 mutant mice after ischemia.**

(A) Genotyping PCR results for WT and HDAC6 mutant mice.

(B) Immunoblot of HDAC6 in the cortex of WT and HDAC6 mutant mice.

(C) Images of representative WT and HDAC6 mutant mice (8 weeks old).

(D) Quantification of body weight of WT and HDAC6 mutant mice. Data shown are independent points and mean; unpaired t-test; n = 6; *p* = 0.353.

(E) Representative image of brains from WT and HDAC6 mutant mice.

(F) Quantification of brain weight. Data shown are independent points and mean; unpaired t-test; n = 6; *p* = 0.499.

(G) Brain slices of indicated genotypes were stained with anti-NeuN antibody (red), and DAPI (blue).

(H) Decreased ischemia-induced brain injury in HDAC6 mutant mice. TTC staining of brain sections from mice of indicated genotypes after ischemic stroke.

(I) Quantification of the lesion volume shown in (H). Data shown are independent points and mean; Unpaired t-test; n = 6; ***, *p* < 0.001.

(J-L) Motor-sensory deficits were evaluated by the modified neurological severity score (mNSS) test (J), foot fault assays (K), and adhesive removal test (L). Data shown are mean ± SEM; two-way ANOVA; n = 6; ***, *p* < 0.001.

Data represent at least three independent experiments.

**Figure S3. MIF was focused on by Bioinformatics analysis.**

(A)Metascape analysis showing bar graph of enriched terms across input gene lists, colored by p-values.

(B) Gene Set Enrichment Analysis (GSEA) results showing regulation of cell death pathway.

**Figure S4. Reduced MNNG-induced DNA fragmentation and cell death with HDAC6 inhibitor or aspirin treatment.**

(A) Neuro2a cells were treated with tubastatin A (10 μM) or aspirin (1 mM) and exposed to MNNG (50 μM) for 15 min. The Neuro2A cells were stained by propidium iodide (red) and Hoechst 33342 (blue) in 12 h after MNNG treatment.

(B) Quantification of cell death shown in (A). Data shown are independent points and mean; one-way ANOVA; n = 3; *, *p* < 0.05; **, *p* < 0.01; ***, *p* < 0.001.

(C-F) Neuro2a cells were treated with tubastatin A (C and D) or aspirin (E and F) and exposed to MNNG (50 μM) for 15 min. Cells were analyzed by immunoblot after 12 h. Quantification of γH2AX level is shown in C and E. Data shown are independent points and mean; one-way ANOVA; n = 9 (D), n = 6 (F);*, *p* < 0.05; ***, *p* < 0.001.

**Figure S5. Attenuated MIF expression in AAV-shMIF-GFP-infected neurons.**

AAV-shNC-GFP and AAV-shMIF-GFP were injected into the cortex of mice. Two weeks after infection, brain sections were stained with anti-MIF antibody. Arrows indicate GFP-positive neurons.

**Figure S6. HDAC6 inhibitor and aspirin reduce MNNG-induced cell death by increasing MIF K78 acetylation.**

(A) MIF mutant Neuro2a cells were transfected with WT or K78R MIF and treated with tubastatin A or aspirin, followed by exposure to MNNG (50 μM) for 15 min. Neuro2a cells were stained with propidium iodide (red) and Hoechst 33342 (blue) 12 h after MNNG treatment.

(B) Quantification of cell death shown in (A). Data shown are independent points and mean; one-way ANOVA; n = 3; *, *p* < 0.05; **, *p* < 0.01.

**Figure S7. Attenuated astrocyte and microglia reactivation in MIF K78Q mice after ischemia.**

(A) Immunoblot analysis of MIF protein in the cortex from WT and K78Q mice.

(B) Quantification analysis of MIF protein levels shown in (A) normalized to GAPDH. Data shown are independent points and mean; n = 6.

(C-F) Ischemia was induced in WT and MIF K78Q mice. Brain sections were stained with anti-GFAP antibody (C) and anti-Iba1 antibody (E). (D and F) Quantification analysis of GFAP (D) and Iba1 (F) intensity. Data shown are independent points and mean; unpaired t-test; n = 6 sections from 3 mice; ***, *p* < 0.001.
